# Non-invasive molecularly-specific millimeter-resolution manipulation of brain circuits by ultrasound-mediated aggregation and uncaging of drug carriers

**DOI:** 10.1038/s41467-020-18059-7

**Published:** 2020-10-01

**Authors:** Mehmet S. Ozdas, Aagam S. Shah, Paul M. Johnson, Nisheet Patel, Markus Marks, Tansel Baran Yasar, Urs Stalder, Laurent Bigler, Wolfger von der Behrens, Shashank R. Sirsi, Mehmet Fatih Yanik

**Affiliations:** 1 Institute of Neuroinformatics, D-ITET, ETH Zurich and UZH, Zurich, Switzerland; 2 Neuroscience Center, Zurich, Switzerland; 3 Department of Chemistry, UZH, Zurich, Switzerland; 4 Department of Bioengineering, UT at Dallas, Richardson, USA

**Keywords:** Neuroscience, Biomedical engineering

## Abstract

Non-invasive, molecularly-specific, focal modulation of brain circuits with low off-target effects can lead to breakthroughs in treatments of brain disorders. We systemically inject engineered ultrasound-controllable drug carriers and subsequently apply a novel two-component Aggregation and Uncaging Focused Ultrasound Sequence (AU-FUS) at the desired targets inside the brain. The first sequence aggregates drug carriers with millimeter-precision by orders of magnitude. The second sequence uncages the carrier’s cargo locally to achieve high target specificity without compromising the blood-brain barrier (BBB). Upon release from the carriers, drugs locally cross the intact BBB. We show circuit-specific manipulation of sensory signaling in motor cortex in rats by locally concentrating and releasing a GABA_A_ receptor agonist from ultrasound-controlled carriers. Our approach uses orders of magnitude (1300x) less drug than is otherwise required by systemic injection and requires very low ultrasound pressures (20-fold below FDA safety limits for diagnostic imaging). We show that the BBB remains intact using passive cavitation detection (PCD), MRI-contrast agents and, importantly, also by sensitive fluorescent dye extravasation and immunohistochemistry.

## Abstract (web summary)

Non-invasive manipulation of brain circuits with molecular and spatial specificity could revolutionize the treatment of brain disorders. Here, the authors remotely concentrate and deliver drugs to focal brain regions without compromising the blood-brain barrier using novel ultrasound sequences and drug carriers.

## Introduction

Central nervous system (CNS) disorders arise from dysfunctions in brain networks involving different cortical, hippocampal, amygdaloidal, striatal, thalamic, and other subfields of the brain, as well as different cell types and molecular targets within these brain regions^1,2^. Despite significant advancements in our understanding of CNS function and pathologies in recent years, translating these findings to therapeutic approaches has been hindered by our inability to selectively manipulate discrete circuits within the brain. Indeed, the most common method of treatment for CNS disorders still remains systemic administration of small molecules. Systemic treatments often cause significant off-target effects at their efficacious dosages by acting in other brain regions or organs/tissues^3,4^, because the receptor-binding sites targeted by most drugs are almost always shared by multiple brain areas, as well as other tissues. This makes it extremely challenging to exclusively modulate pathological networks. Current methods under development to target specific brain circuits such as transcranial magnetic stimulation (TMS) or penetrating electrodes (i.e., deep-brain stimulation; DBS) lack cellular and molecular specificity while also either have low spatial resolution, or are invasive^5^.

To address this challenge, we developed a unique spatially-targeted and molecularly-specific drug delivery technology by using novel focused ultrasound (FUS) sequences and ultrasound-sensitive drug carriers. First introduced in the 1940s^6^, FUS allows noninvasive delivery of mechanical energies deep into the tissue and even through the human skull with millimeter-resolution^7^. FUS by itself lacks cellular and molecular selectivity^7^. However, FUS has been combined with microbubbles to open the blood–brain barrier (BBB) locally to deliver molecules that otherwise do not cross the intact BBB^8–10^. This is a promising development for acute delivery of macromolecules and has significant potential, particularly in the treatment of genetic disorders when used along with the pioneering FUS-mediated gene delivery approaches^11–15^. FUS technology’s safety for one-time (or twice) controlled BBB opening has been clinically tested recently^16^. Despite considerable promise, FUS-mediated BBB opening might cause cellular damage, significant immune response, and blood cell infiltration to the brain, where the damage severity and type depends on the choice of FUS parameters^17,18^. In many neurological and neuropsychiatric disorders, lifelong drug treatment is needed, and any sort of repeated (chronic) BBB opening for drug delivery might have severe consequences. Importantly, compromise of BBB and vasculature is associated with the onset and progression of various neurological and neurodegenerative disorders^19,20^. Hence, while FUS-mediated BBB opening might be acceptable for one-time acute delivery of viral vectors or other macromolecules, it is currently unclear what risks repeated BBB opening may pose for long-term treatment of neurological and neuropsychiatric disorders. Furthermore, since BBB opening on its own modulates neuronal activity and behavior in rodents and primates^21–23^, it cannot be used in circuit investigations without confounding effects.

Most clinically approved neurological and neuropsychiatric drugs are small molecules that already cross the BBB on their own. However, these drugs rather need to be prevented from crossing the BBB, except within the targeted brain area(s), to avoid undesirable off-target effects. Recently, FUS-mediated small-molecule delivery has been explored in animal models, however, these approaches either require BBB opening or are too inefficient^10,21,24^ (see “Discussion”).

**Fig. 1:**
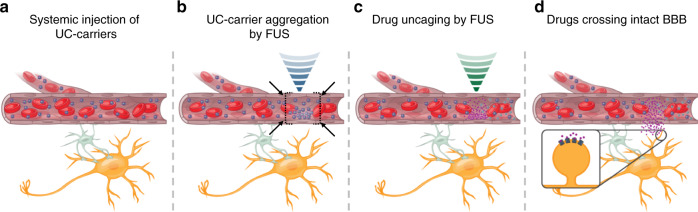
Concept of focal aggregation and uncaging of ultrasound-controlled drug carriers. **a** UC-carriers are continuously infused (blue particles within the capillary) intravenously, which circulate stably. **b** UC-carriers are first aggregated locally by acoustic radiation forces generated by ultrasound waves (blue). **c** The locally concentrated UC-carriers are then uncaged to release drugs using acoustic uncaging forces produced by ultrasound waves (green). **d** Small molecules diffuse across the intact BBB reaching their molecular targets (ultrasound beam is not to scale).

Here, we demonstrate an approach to deliver small molecules with millimeter-precision, without opening BBB, by focally aggregating drugs with orders-of-magnitude greater efficiency relative to systemic delivery. To achieve this, we developed stable drug-loaded liposomes tethered to microbubbles (Ultrasound-Controlled drug carriers; UC-carriers) and a unique multi-component Aggregation-Uncaging FUS (AU-FUS) sequence. We first systemically inject UC-carriers containing small-molecule drugs into rats (Fig. 1a). These small-molecule cargos can be existing FDA-approved neurological or neuropsychiatric drugs, which are already capable of crossing BBB but otherwise remain in the circulation while encapsulated in UC-carriers. Next, we use ultrasound waves, to aggregate the UC-carriers (Aggregation sequence, Fig. 1b) at the desired regions in the brain. After local aggregation, we uncage the drugs from the UC-carriers with a second ultrasound pulse sequence (Uncaging sequence, Fig. 1c), releasing these small molecules into the blood stream. The small molecules then readily cross the intact BBB within the focal area, to reach their molecular targets (Fig. 1d). Our ability to enhance focal delivery by aggregation is critical to prevent off-target effects because the remaining drug carriers in the blood are eventually cleared, releasing their drug cargo systemically.

We developed and validated our approach in vivo by non-invasively modulating the propagation of neuronal activity through a defined cortical microcircuit, specifically the rodent vibrissae sensory-motor pathway^25,26^. We manipulate this circuit by focally inhibiting vibrissa sensory cortex, through ultrasound-mediated uncaging of the GABA_A_ receptor agonist, muscimol. We demonstrate that there is no detectable BBB opening or damage using sensitive techniques. We also demonstrate by electrophysiology that the released drug affects only the targeted area. As we focally aggregate UC-carriers, our approach reduces the administered muscimol dose by up to three orders of magnitude.

## Results

### UC-carriers and AU-FUS sequence

**Fig. 2:**
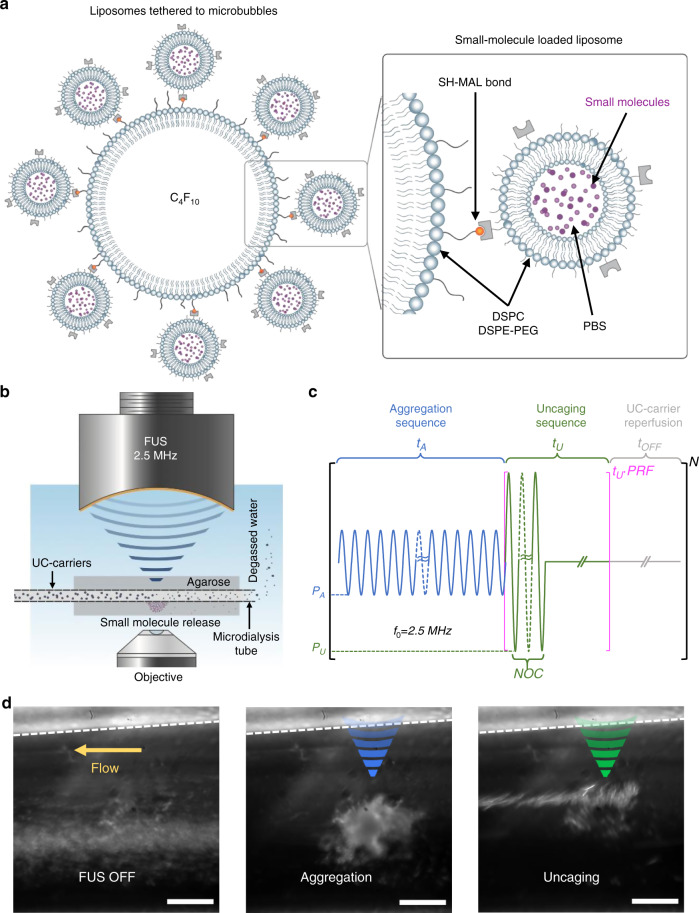
Focal Aggregation and Uncaging of small molecules by AU-FUS. **a** Small-molecule-loaded Ultrasound-Controlled carrier (UC-carrier) design. DSPC and DSPE-PEG2k form the lipid shells of the microbubbles (monolayer) and liposomes (bilayer). The lipid microbubbles (~1.5 μm mean diameter) have perfluorobutane (PFB, C_4_F_10_) gas core and have DSPE-PEG5k-Mal on the surface for conjugation with liposomes (~116 nm mean diameter; conjugated UC-carriers have ~1.7 μm mean diameter; Supplementary Fig. 3), which contained DSPE-PEG5k-SH on the surface, with a PBS core (illustration modified from Wang et al.^69^). Small molecules are actively loaded into the liposomes using repeated freeze-thaw cycles, prior to conjugation (see “Methods”). **b** Setup for in vitro characterization of FUS sequences consists of dye-loaded UC-carriers flowing through microdialysis tubing (13 kDa cutoff, single pass), embedded in low-melt agarose. The system is confocally aligned to an inverted water-immersion 60x objective lens, and a 2.5 MHz FUS transducer. The entire setup is inside a custom-made water tank, filled with degassed Milli-Q water. **c** The AU-FUS sequence design. First, the aggregation sequence with peak-negative pressure *P*_*A*_ and duration *t*_A_ is applied. This is immediately followed by the uncaging sequence with peak-negative pressure *P*_U_ and with a fixed number of cycles (NOC) and pulse-repetition frequency (PRF) for a total duration *t*_U_. A reperfusion period (FUS-OFF) with duration *t*_OFF_ permits reperfusion of UC-carriers. The entire sequence is repeated for the duration of sonication (*N*). **d** UC-carriers (white particles in the image) flowing through microdialysis tubing (left) with no FUS. Aggregation (blue, middle) and uncaging (green, right) sequences concentrate the UC-carriers focally and release the small molecules, respectively (see also Supplementary Movie 1). Dashed line indicates wall of tubing. Scale bar is 50 μm.

We tethered drug-encapsulated liposomes to ultrasound-sensitive microbubbles (with perfluorocarbon gas core) using thiol-maleimide chemistry (Fig. 2a) to create UC-carriers, modified from earlier studies^27,28^. We used this configuration because tethering drug-loaded liposomes to microbubbles makes liposomes indirectly responsive to ultrasound, allowing spatial and temporal control of drug deposition. The use of liposomes also allows encapsulation of diverse small molecules using either the hydrophilic liquid core or the lipophilic hydrocarbon shell. We prepare UC-carriers by loading either sodium fluorescein dye (model drug for in vitro experiments) or muscimol (for in vivo experiments) into the core of the liposomes.

To characterize the behavior of UC-carriers and to optimize FUS sequences, we built a custom in vitro setup which consists of a microdialysis channel embedded in agarose^29^. This channel was confocally aligned to an inverted microscope objective lens and FUS transducer. The entire setup was enclosed in a custom-built water tank, filled with degassed Milli-Q water (Fig. 2b). Fluorescein-loaded UC-carriers flowed continuously through the dialysis tubing at velocities (10 μL min^−1^, corresponding to 5 mm s^−1^ of flow velocity) that mimicked the highest blood flow rates in brain capillaries^30^. The carriers were then exposed to either commonly used BBB opening FUS (standard-FUS sequences) or our AU-FUS sequences. We measured the model drug (fluorescein dye) release into the agarose both within the focus and outside the focus of the FUS transducer.

We began with standard-FUS ultrasound sequences (single-component burst pulse sequence, Table 1), as well as multi-component sequences^29,31^. Ferrara and colleagues^29^ used primary radiation forces to push lipospheres towards capillary walls to enhance drug delivery. They tried to avoid aggregation of microparticles caused by secondary radiation forces (see “Discussion”) for their purposes. In contrast, we hypothesized that microparticle aggregation could itself be useful to enhance focal drug release into the blood stream if we can aggregate microparticles locally by orders of magnitude. Indeed, after several rounds of in vitro optimizations, we developed AU-FUS sequences where the first component was able to trap and aggregate the UC-carriers (Fig. 2c-blue, Fig. 2d middle, see also Supplementary Movie 1), while the second component, an uncaging sequence, was able to release the drug payload (Fig. 2c-green, Fig. 2d right, see also Supplementary Movie 1). Importantly, our AU-FUS sequences not only delivered more small molecules than the standard-FUS sequences, but also required several-fold lower ultrasound pressures than both the standard-FUS sequences and the reported fragmentation pressures of Ferrara and colleagues (Supplementary Fig. 1a, Supplementary Movie, and Table 1). Even doubling the pressures of standard-FUS sequences did not result in greater deposition (Supplementary Fig. 1a), suggesting the acoustic radiation forces generated by the first component of our AU-FUS sequences were important for efficient uncaging. We also characterized the effects of individual components of AU-FUS sequences using our in vitro setup. We observed that neither aggregation nor uncaging sequences alone were effective in releasing small molecules and that both components are essential for low-pressure drug delivery (Supplementary Fig. 1b). The magnitude of drug deposition in vitro we obtained is substantial considering that the microdialysis tubing is about 20 times larger than the brain capillaries and the in vitro flow rate we used is 5 times the average rate of blood flow in the brain capillaries^32^, thus rapidly washing away most of the uncaged drug before it reaches the tubing walls.

**Table 1:** FUS parameters.

Condition	*P*_A_ (MPa)	*t*_A_ (ms)	*P*_U_ (MPa)	*t*_U_ (ms)	*t*_OFF_ (ms)	PRF (Hz)	NOC
standard_1_-FUS	1.25	-	-	-	-	1	25,000
standard_2_-FUS	2.5	–	–	–	–	1	25,000
standard_3_-FUS	0.75*	–	–	–	–	1	25,000
standard_4_-FUS	1.5*	–	–	–	–	1	25,000
AU_1_-FUS (in vitro)	0.3	500	0.5	90	300	30	10,000
AU_2_-FUS (in vitro)	0.38	500	0.63	90	300	50	10,000
AU_3_-FUS (in vitro)	0.25	1000	0.63	90	300	100	1000
AU-FUS_in-vivo_	0.075*	1000	0.188*	90	300	100	1000

*P*_A,_ *P*_U_—peak-negative pressure in megapascal (MPa) for aggregation and uncaging sequences, respectively (*accounting for ~70% skull attenuation; see “Methods” and Supplementary Figs. 4 and 5), *t*_A_—pulse duration in milliseconds (ms) of aggregation sequence, *t*_U_—duration in milliseconds (ms) of uncaging sequence, *t*_OFF_—duration in milliseconds (ms) of delay between the end of the uncaging sequence and the start of the following aggregation sequence, *PRF*—pulse-repetition frequency in Hz, *NOC*—number of cycles.

**Fig. 3:**
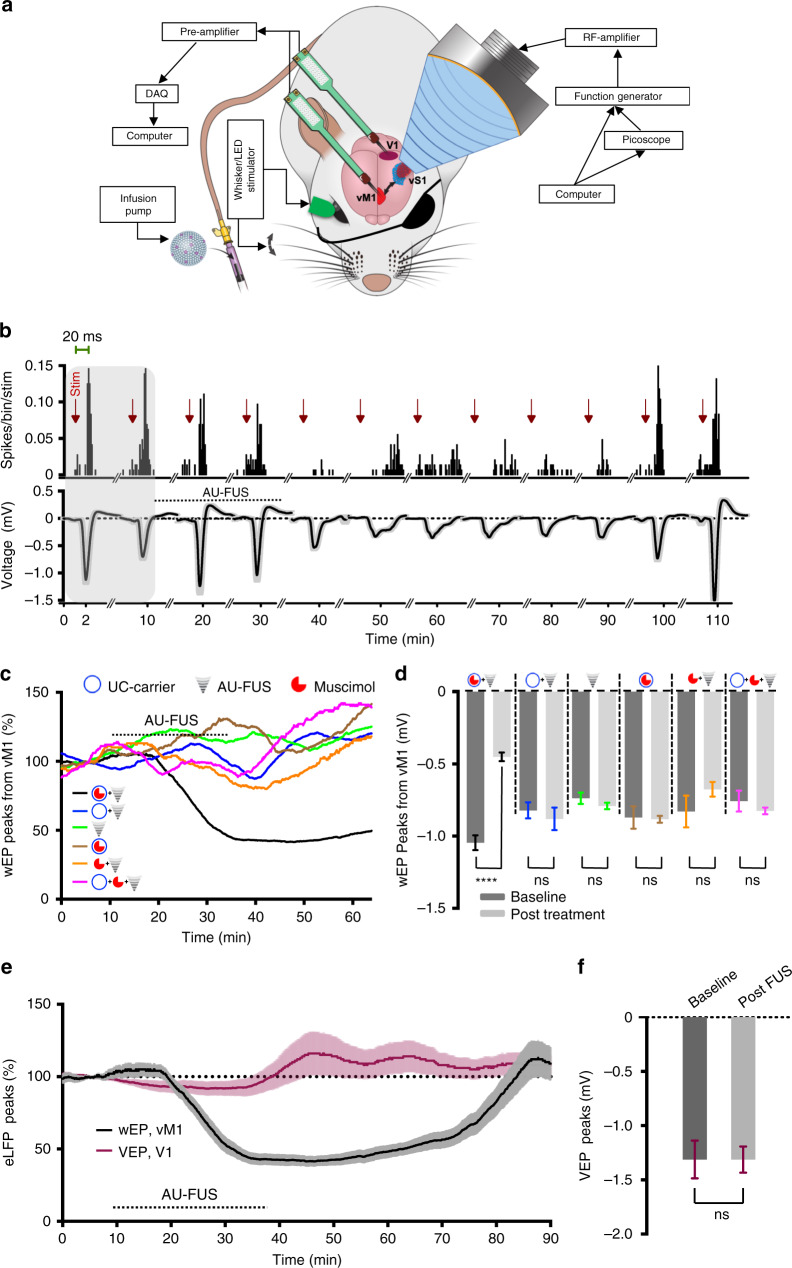
Receptor-specific focal modulation of cortical circuits by AU-FUS. **a** Experimental setup for in vivo drug delivery. The recording probe was inserted in vM1 or V1 and the FUS transducer was positioned above vS1. Contralateral whiskers (eye) were mechanically deflected (visually stimulated) at 0.3 Hz. UC-carriers were IV-injected through the tail vein. vM1 and V1 recordings were performed on separate cohorts. **b** Representative peri-stimulus histograms (PSTHs, top, bin size 2 ms) and wEPs (bottom) upon focal aggregation of muscimol-loaded UC-carriers and uncaging of muscimol from one experiment. Following a baseline recording (shaded area), the animal was injected with muscimol-loaded UC-carriers and FUS was turned on. wEPs showed 62.62% inhibition. PSTHs and wEPs recovered completely ~75 min post FUS. The wEPs and PSTHs were averaged over 2 min windows. **c** Time course of normalized wEPs in vM1. Muscimol-loaded UC-carriers with AU-FUS [black line, *n* = 24 (6 rats × 4 recording sites)], vehicle-loaded UC-carriers with AU-FUS [blue line, *n* = 24 (6 rats × 4 recording sites)], AU-FUS without UC-carrier injection [green line, *n* = 36 (9 experiments × 4 recording sites, from 5 rats)], muscimol-loaded UC-carrier injection without AU-FUS [brown line, *n* = 20 (5 rats × 4 recording sites)], systemic injection of free muscimol (250 ng) with AU-FUS [orange line, *n* = 20 (5 rats × 4 recording sites)], systemic injection of free muscimol (250 ng) and vehicle-loaded UC-carriers with AU-FUS [pink line, *n* = 20 (5 rats × 4 recording sites)]. **d** Post-treatment (30 min average) vs. baseline (10 min average) wEPs. Statistical comparison (Wilcoxon matched-pairs-signed rank test): baseline vs. treatment; Black (muscimol-loaded UC-carriers + AU-FUS), ****p < 0.0001; Blue (vehicle-loaded UC-carriers + AU-FUS), *p* = 0.3902; Green (AU-FUS only), *p* = 0.2371; Brown (muscimol-loaded UC-carriers only), *p* = 0.8695; Orange (250 ng free muscimol + AU-FUS), *p* = 0.1737; Pink (vehicle-loaded UC-carriers + 250 ng free muscimol + AU-FUS), *p* = 0.2611. All data is mean ± s.e.m. **e** Locally uncaged muscimol does not spread to neighboring cortical areas. Normalized eLFP responses (VEP, purple line) in neighboring V1, as compared to responses (wEP, black line) in vM1. **f** VEPs from V1. Statistical comparison (Wilcoxon matched-pairs-signed rank test): baseline (10 min average) vs. treatment (30 min average), (*p* = 0.5199, *n* = 16 (4 rats × 4 recording sites). All data is mean ± s.e.m.

**Fig. 4:**
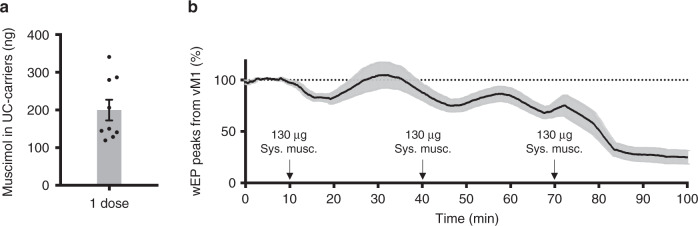
Focal 1300-fold enhancement of drug response by AU-FUS compared to systemic levels. **a** LC-HR-MS/MS quantification of muscimol loaded in one dose of AU-FUS treatment. All data is mean ± s.e.m, showing all points. Average = 199.7 ng, *n* = 9. **b** Time course of wEPs (negative peak) in vM1 during systemic injection of muscimol (“Sys. musc.”). Following 10 min of baseline, 130 μg of muscimol (~650 times single muscimol-loaded UC-carrier injection dose) is manually injected over 1 min, every 30 min (black arrows). Data is plotted as the moving average (window size = 180 whisker deflections). All data is mean ± s.e.m. *n* = 16 (4 rats × 4 recording sites). Equivalent inhibition by AU-FUS would occur between 260 and 390 μg systemic injection.

**Fig. 5:**
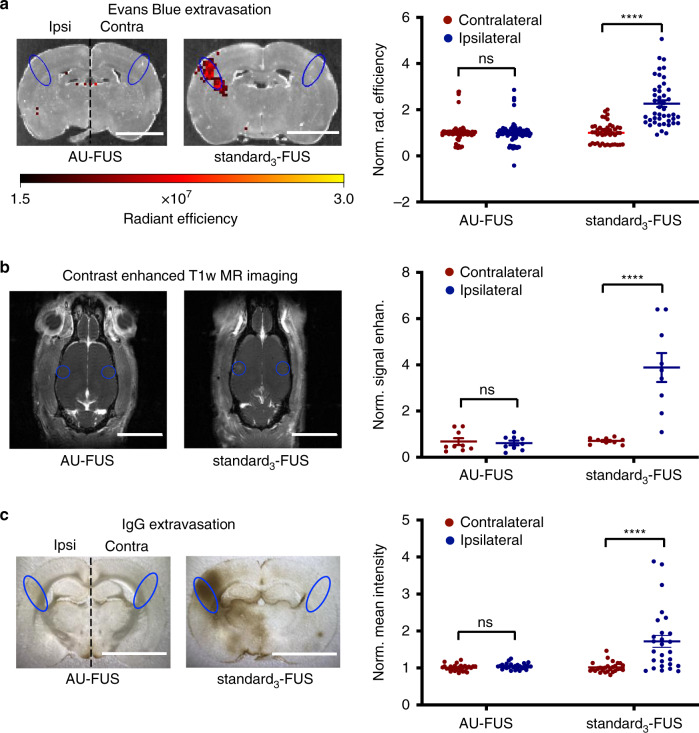
Preservation of blood–brain barrier integrity following AU-FUS. **a** Evans Blue extravasation. Evans Blue was IV-injected. Representative IVIS spectrum images, at the FUS target location (vS1) following AU-FUS (muscimol-loaded UC-carriers with FUS, left image) vs. standard_3_-FUS (right image). Regions of interests (ROIs) (1.5 ×3.5 mm, blue) were measured as radiant efficiency ipsilateral to FUS application and were compared to the contralateral vS1 (scale bar: 0.5 cm). Radiant efficiency values within ROIs for AU-FUS and standard_3_-FUS sequences were quantified [*n* = 90 (6 rats × 15 brain sections) for AU-FUS; same rats as in Fig. 3c; *n* = 45 (3 rats × 15 brain sections) for standard_3_-FUS]. Pairwise Mann–Whitney rank-sum test AU-FUS (ipsilateral vs. contralateral, *p* = 0.6577), standard_3_-FUS (ipsilateral vs. contralateral, ****p < 0.0001). **b** MRI contrast agent extravasation. Animals were injected with Omniscan (Gd) and imaged. Representative brain images at the FUS target location (vS1) after AU-FUS (left image) compared to standard_3_-FUS sequence (right image). ROIs (1.0 ×1.0 mm, orange, approximate ROI location) were measured as signal enhanced T1-weighted MR images, ipsilateral to FUS application, which were compared to the contralateral vS1 (scale bar: 1.0 cm). See Supplementary Fig. 9 for zoomed images. Baseline-subtracted contrast-enhanced T1-weighted MR image ROIs using AU-FUS and standard_3_-FUS sequences were quantified [*n* = 9 (3 rats × 3 brain sections) for AU-FUS and standard_3_-FUS]. Pairwise Mann–Whitney rank-sum test, AU-FUS (ipsilateral vs. contralateral, *p* = 0.9494), standard_3_-FUS (ipsilateral vs. contralateral, ****p < 0.0001). **c** IgG immunohistochemistry. Photographic images of immunohistochemical IgG staining of brain sections from the center of the focal volume following AU-FUS (left photo) vs. standard_3_-FUS (right photo) (scale bar: 0.6 cm). Normalized mean intensity values from ROI analysis of brightfield images of brain sections stained for IgG were quantified. Intensities were obtained from ROIs (1.5 × 3.5 mm) contralateral and ipsilateral to FUS treatment within the FUS focal volume [*n* = 27 (3 rats × 9 sections) for AU-FUS; *n* = 27 (3 rats × 9 sections) for standard_3_-FUS] and normalized with the average contralateral mean intensity. Pairwise Mann–Whitney rank-sum test AU-FUS (ipsilateral vs. contralateral, *p* = 0.0970), standard_3_-FUS (ipsilateral vs. contralateral, ****p < 0.0001).

**Fig. 6:**
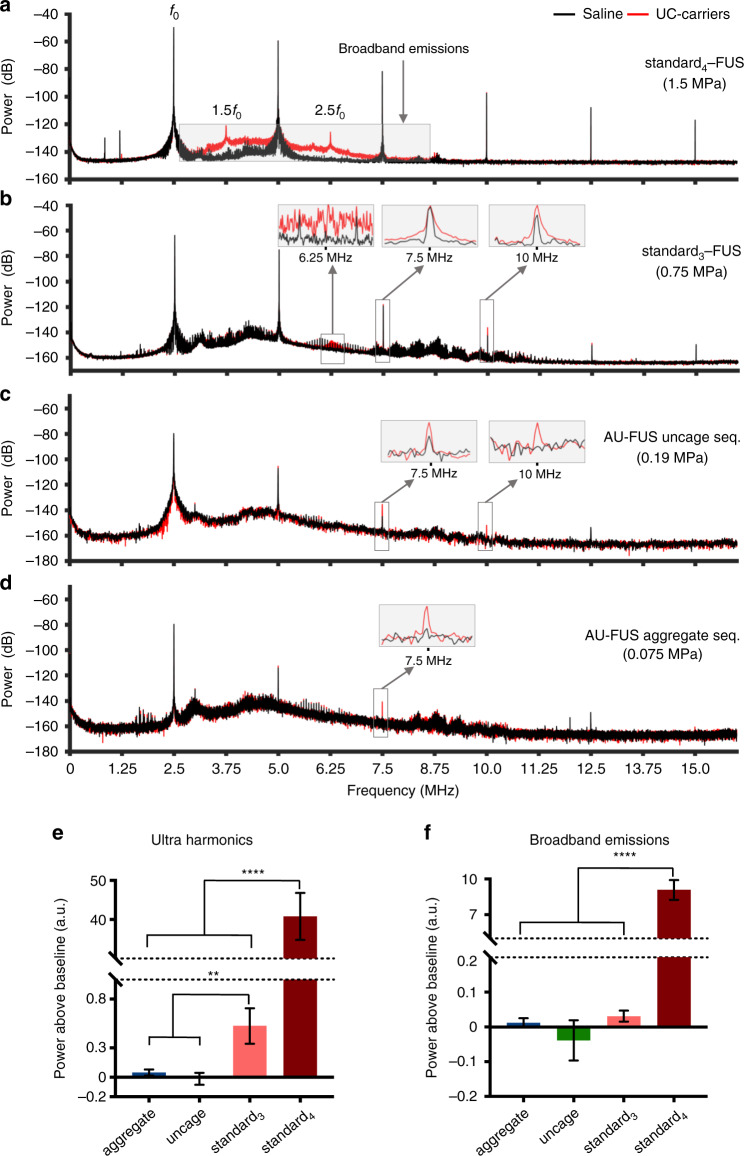
Passive cavitation detector response shows no signature of BBB opening in vivo. **a** Fast Fourier Transform (FFT) analysis of representative responses to standard_4_-FUS sequence. Broadband emissions (shaded box) and ultra-harmonics (3.75 and 6.25 MHz) are detected only in the presence of UC-carriers (red) as compared to saline (black), indicating the inertial cavitation regime of BBB opening (see Supplementary Fig. 12). **b** FFT analysis of representative response to standard_3_-FUS sequence. Ultra-harmonics but not broadband emissions (signatures of stable cavitation^46^) are detected only in the presence of UC-carriers (red) as compared to saline (black), consistent with stable-cavitation regime of BBB opening (see Supplementary Fig. 12). Insets show ultra-harmonics and integer harmonics. Data is mean of 50 pulses (**a**, **b**). **c** FFT analysis of representative response to AU-FUS uncaging sequence (preceded by AU-FUS aggregation sequence) showing neither ultra-harmonics nor broadband emissions in the presence of UC-carriers (red) vs. saline (black), consistent with no BBB opening (see Supplementary Fig. 12). Insets show integer harmonics. Note that the FFT data has less samples due to 0.4 ms pulse width vs 2 ms in others. **d** FFT analysis of representative response to AU-FUS aggregation sequence (preceding the AU-FUS uncaging sequence) showing neither ultra-harmonics nor broadband emissions in the presence of UC-carriers (red) vs. saline (black), suggesting no BBB opening (see Supplementary Fig. 12). Insets indicates integer harmonics. Data is mean of 7 pulses (**c**, **d**). **e** Calculation of ultra-harmonics above baseline [*n* = 150 (3 rats × 50 pulses) for standard_3,4_-FUS, *n* = 21 (3 rats × 7 pulses) for AU-FUS sequence]. All data is mean ± s.e.m. One-tailed, unpaired *t*-test with Welch’s correction, aggregate seq. vs. standard_3_-FUS, **p = 0.0052; uncage seq. vs. standard_3_-FUS, **p = 0.0026; aggregate seq. vs. standard_4_-FUS, ****p < 0.0001; uncage seq. vs. standard_4_-FUS, ****p < 0.0001; standard_3_-FUS vs. standard_4_-FUS, ****p < 0.0001. **f** Calculation of broadband emissions above baseline [*n* = 150 (3 rats × 50 pulses) for standard_3,4_-FUS, *n* = 21 (3 rats × 7 pulses) for AU-FUS sequence]. All data is mean ± s.e.m. One-tailed, unpaired *t*-test with Welch’s correction, aggregate seq. vs. standard_4_-FUS, ****p < 0.0001; uncage seq. vs. standard_4_-FUS, ****p < 0.0001; standard_3_-FUS vs. standard_4_-FUS, ****p < 0.0001. Pressures account for skull attenuation (see Table 1). See Supplementary Fig. 11 for PCD setup.

However, when tested in vivo (with FUS parameters adjusted to account for the attenuation of ultrasound waves by the skull; see “Methods”), even our lowest-power in vitro optimized AU-FUS parameters [AU_1_-FUS (in vitro)] still caused weak BBB opening (Supplementary Fig. 2). Since neither in vitro deposition nor artificial BBB models sufficiently mimic in vivo BBB, capillaries, drug, and blood–plasma interactions, we further optimized our AU-FUS pulse sequences by iterations between in vivo and in vitro experiments (the sizes of FUS transducers and the objective lenses make it infeasible to image microparticles in brain capillaries during FUS). We systematically varied many parameters, including the number of cycles, pulse-repetition frequencies, amplitudes for each component of AU-FUS, pulse-to-pulse delays (FUS-OFF period), UC-carrier concentrations, and UC-carrier lipid chemistries and compositions. We finally identified a novel in vivo AU-FUS sequence (AU-FUS_in-vivo_) that was able to deliver small molecules with high efficiency while also completely avoiding BBB damage (in vivo experiments shown in Figs. 3–6).

### Receptor-specific modulation of a cortical circuit in vivo

We tested our AU-FUS sequence in vivo (AU-FUS_in-vivo_; see Table 1) by manipulating a specific cortical network without opening BBB: Rat vibrissa motor cortex (vM1) receives whisker sensory information (~80%) through projections from vibrissa sensory cortex (vS1, “barrel cortex”)^33^. The vS1-vM1 circuit is a good model for in vivo study of FUS-mediated delivery of drugs as vS1 and vM1 are anatomically distant, yet strongly connected brain areas. This allowed us to sonicate vS1 site while doing electrophysiological recordings in vM1 to assess response of drug delivery to vS1, thus eliminating two major confounding effects: (1) Avoiding BBB opening in vS1 due to electrode insertion, (2) Avoiding possible mechanical displacement of electrodes because of FUS pressure. It was also possible to record from another sensory cortical area (V1) in close proximity to vS1 to test potential spread of drug after uncaging.

When rodent whiskers are mechanically deflected, evoked activity propagates from brainstem via thalamus to vS1, and then to vM1. During simultaneous recordings we observed the peaks of whisker-evoked potentials (wEPs) first in vS1 and ~3.0 ms later in vM1 (Supplementary Fig. 6), consistent with the latency of the spiking response reported previously^33^. We tested whether inhibiting vS1 by our technique would suppress wEPs in vM1 in anesthetized rats. We positioned the FUS transducer above the intact skull and focused it on vS1. Using a multielectrode array, we monitored the wEPs online and the whisker-evoked multi-unit activity in vM1 (Fig. 3a). In parallel, we continuously intravenously (IV) injected muscimol-loaded UC-carriers, focally aggregated them, and subsequently uncaged muscimol by applying our AU-FUS sequence repeatedly on vS1. Muscimol which is an agonist of ionotropic GABA_A_ receptors, the major receptor responsible for fast inhibitory transmission in the brain, readily crosses BBB^34^.

Fig. 3b shows that AU-FUS-mediated delivery of muscimol inhibits vS1 and reduces the whisker evoked multi-unit activity and wEPs in vM1 (i.e. vS1’s projection target). Overall, after AU-FUS-mediated inhibition of vS1, the amplitudes of wEPs in vM1 were strongly reduced by 56.02 ± 3.37% (black line in Fig. 3c, d), and it took ~50 mins on average to return to baseline activity. We performed a complete set of control experiments in order to confirm the specificity of our approach and also verified that only muscimol-loaded UC-carriers under AU-FUS_in-vivo_ sequence results in local inhibition. These controls were: (1) AU-FUS application with vehicle-loaded UC-carriers (blue line in Fig. 3c, d), (2) AU-FUS application without UC-carrier injection (green line in Fig. 3c, d), (3) muscimol-loaded UC-carriers without AU-FUS application (brown line in Fig. 3c, d), (4) systemic injection of free muscimol with AU-FUS application (orange line in Fig. 3c, d), (5) systemic injection of free muscimol and vehicle-loaded UC-carriers with AU-FUS application (pink line in Fig. 3c, d). We did not observe any statistically significant changes in the wEPs in vM1 under any of the 5 control conditions. Therefore, the reduction of wEPs in vM1 cannot be attributed to nonspecific effects of the AU-FUS or the UC-carriers.

To rule out that the observed modulation of vM1 could be due to spreading of muscimol from vS1, we recorded from a cortical area close to vS1 but without significant connectivity with vS1: We delivered muscimol to vS1 using AU-FUS and measured visually evoked potentials (VEPs) from primary visual cortex (V1) (Fig. 3a). We did not observe any statistically significant changes in VEPs recorded from V1 (purple, Fig. 3e, f), in spite of the shorter distance between vS1 and V1 (4 mm for vS1-V1 vs. 7 mm for vS1-M1 in rats). Our experimental results are also consistent with our initial estimates that small molecules cannot be spreading large distances to induce physiological responses: Spreading could happen through two different means. First, muscimol after uncaging could perfuse through the capillaries to distant regions. Assuming the diffusion coefficient of a small-molecule in water as *D* = 1.5 × 10^−5^ cm^2^ s^−1^, and a capillary radius of *r* = 5 μm, small molecules would take *τ* = 16 ms (*τ* = *r*^2^*/D*) on average to reach to the capillary walls from anywhere within the capillary. Assuming an average blood flow speed in capillaries of *v* = 1.5 mm s^−1^, we expect free muscimol to flow only ~25 μm (*λ* = *τ* • *v*) beyond its release site before it reaches the blood–brain barrier. Even if muscimol after uncaging does not enter the brain tissue immediately and remains in the circulation, it cannot reach far away tissues before entry to veins because the maximal length of capillaries in the rat brain is only about 250 μm^35^ and, drug uptake to the brain is mainly confined to the capillaries^36^. Importantly, muscimol’s concentration will be negligible after entering the vein and redistributing in systemic circulation^34^. Second, muscimol could diffuse within the interstitial space. The diffusion coefficient of muscimol in rat barrel cortex is *D* = 8.7 × 10^−6^ cm^2^ s^−1^ (ref. ^37^). Given that the maximum inhibition in vM1 occurs within 20 min (Fig. 3c), muscimol can diffuse in tissue only up to ~1 mm. This diffusion distance has been confirmed by measuring the spread of fluorescent muscimol in the rat brain tissue^38^. In deeper subcortical structures, the observed rostrocaudal spread of muscimol has been shown to be ~1.7 mm on timescales comparable to muscimol action in our work^39^. Since vM1 is ~7 mm away from vS1 in rats, muscimol diffusion in the tissue from release site to recording site cannot be the cause of observed neuronal inhibition. Additionally, radial diffusion of muscimol also rapidly dilutes muscimol (~*d*^3^) with distance (*d*) from the delivery locus, thus making its concentration too low to induce physiological responses. These estimates support our experimental finding above. Thus, AU-FUS drug delivery is highly local and the observed neuromodulations cannot be due to the spreading of drug after uncaging.

### Focal 1300-fold enhancement of drug response by AU-FUS

We determined the total amount of muscimol we inject in a single dose of UC-carriers for AU-FUS treatment to be ~200 ng using liquid chromatography-high-resolution tandem mass spectroscopy (LC-HR-MS/MS) (Fig. 4a). We tested the effect of systemically administered muscimol on the wEPs in vM1 (without AU-FUS or UC-carriers). A comparable reduction of wEPs was observable only after systemically administering at least 1300 times the measured payload of our UC-carriers (at least 260 μg systemic muscimol, Fig. 4b). This is consistent with the amount of systemic muscimol required for brain inactivation, which is ~1.6 mg kg^−1 ^^40,41^.

### Blood–brain barrier integrity and normothermia preservation

We assessed BBB integrity to determine the safety of our sequence, as BBB opening can be accompanied by inflammation and cellular damage. We evaluated BBB opening by measuring the extravasation of Evans Blue dye (EB) through In Vivo Imaging System (IVIS) spectrum epifluorescence imaging, Gadolinium (Gd)-enhanced T1-weighted Magnetic Resonance (MR) imaging, and extravasation of Immunoglobulin G (IgG) by immunohistochemical staining, none of which easily crosses the intact BBB. We measured tracer (EB, Gd, and IgG) extravasation in regions of interest (ROIs) ipsilateral and contralateral to AU-FUS in vS1 to determine BBB opening. There was no statistically significant difference in EB or IgG extravasation (Fig. 5a, c and Supplementary Fig. 7a), or Gd contrast enhancement (Fig. 5b and Supplementary Fig. 7b) when comparing ROIs contralateral and ipsilateral to AU-FUS treatment site for the animals undergoing AU-FUS independent of whether tracer (EB and Gd) was injected before or after sonication. In contrast, marked EB, Gd, and IgG labeling demonstrated profound BBB opening with standard_3_-FUS parameters (see Table 1) (Fig. 5). See Supplementary Fig. 8 for entire focal volume range (EB). See Supplementary Fig. 9 for zoomed contrast-enhanced magnetic resonance imaging (MRI) image for better visualization.

Since it is known that FUS can cause rapid temperature increases in the brain^42^, which can have adverse effects on BBB, we monitored the temperature within our AU-FUS focal volume. We measured an average temperature increase of only 0.12 °C during the sonication (Supplementary Fig. 10), which is within the normal range of temperature fluctuations in the awake behaving rats and is significantly below the threshold for alterations in BBB permeability, cellular damage, or changes in cell activity^43,44^.

### PCD spectra reveal no signature of BBB opening with AU-FUS

We measured passive cavitation responses of UC-carriers under standard-FUS sequences at different pressures, and under our AU-FUS_in-vivo_ sequence. Upon injection of UC-carriers, the standard_4_-FUS sequence (1.5 MPa) caused strong broadband emissions (i.e., inertial cavitation^45,46^), along with ultra-harmonic emissions (Fig. 6a, e, f). This is a harmful BBB opening regime where microbubbles violently collapse, causing shock waves and microjet streams, leading to tissue damage and cell death along with BBB opening^47,48^. Sonicating with the standard_3_-FUS sequence (0.75 MPa) caused ultra-harmonic emissions with negligible broadband emissions (stable cavitation^46^) (Fig. 6b, e, f) along with Evans Blue extravasation, indicating BBB opening. However, sonication of UC-carriers with our AU-FUS sequence caused neither ultra-harmonics nor broadband emissions (Fig. 6c–f). As expected, we only saw an increase in the amplitudes of integer harmonics upon injection of UC-carriers. In this regime, we do not see any BBB opening after extensive histological analysis (Fig. 5).

## Discussion

Technological advancements that enable safe and robust manipulation of specific neural circuits involved in disease pathologies can address both efficacy and molecular specificity challenges of existing treatments. In this study, we demonstrate a novel technique that allows efficacious and non-invasive modulation of specific brain circuits by spatially targeted delivery of receptor-specific small molecules. We overcame several fundamental challenges to make this possible: (1) Devising a means to trap and concentrate sufficiently high numbers of UC-carriers in circulation, (2) Uncaging ample amounts of drug with minimal ultrasound energies to induce significant physiological responses, (3) Avoiding opening/damaging BBB and tissue heating, (4) Producing stable microbubble-liposome complexes suitable for use with multi-component FUS, which can carry sufficient payloads with diverse physical properties, and (5) Identifying FUS sequences that do not cause nonspecific neuronal responses (i.e., without molecular specificity) due to the application of FUS alone (i.e., in the absence of UC-carriers).

Interestingly, our AU-FUS sequences seem to concentrate the drug carriers not only along the radial axis of the capillaries (primary radiation forces), but also along the longitudinal axis of the capillaries (secondary radiation forces). The aggregation component of our AU-FUS sequence likely utilizes secondary radiation forces, known as “Bjerknes forces”, to drive bubbles into close proximity of each other at low pressures^49^. The dynamics of microbubble aggregation is complex as they continue to volumetrically oscillate, coalesce, break up, and re-form repeatedly under continuous low-intensity ultrasound^50^. In addition, our liposome-loaded microbubbles likely respond differently to FUS compared to microbubbles alone. The application of higher intensity uncaging pulses in the second part of the AU-FUS sequence may promote better drug release from the liposomes in microbubble clusters compared to individual microbubbles, since gas from the microbubbles would leak out at high velocities, as suggested by Klibanov and colleagues^51,52^, significantly enhancing the shear effects on nearby liposomal bilayers in dense microbubble clusters, thereby destabilizing and releasing liposome contents, as shown by Marmottant et al.^53^.

A major challenge in the chronic use of ultrasound-mediated drug release in the brain has been that previous approaches either required or caused unavoidable BBB opening^10,21^. Recently it has been shown that BBB opening can induce sterile inflammation^18^ under certain conditions, has strong effects on cell activity and behavior^21–23^, and is implicated in neurodegenerative diseases^19,20^. Therefore, for use in chronic treatments of many brain disorders, it is vital to achieve FUS-mediated drug release without compromising BBB as we demonstrated here.

Recent studies by Airan et al.^24,54,55^ suggested that FUS-sensitive perfluoropentane (PFP) nanoemulsions (instead of the microbubbles+liposomes used here) can allow delivery of lipophilic-only compounds to the brain without opening BBB. While highly interesting, these methods require extremely large amounts of encapsulated drug to be injected to observe any effects (nanoemulsions containing 1 mg kg^−1^ propofol need to be injected to show similar effects to systemic injection of only 2 mg kg^−1^ propofol). As the local propofol concentration required for inhibition is miniscule (tens of nanograms^56^), this suggests that most of the propofol content of the nanoemulsions must be released nonspecifically to the rest of the brain, which is in fact a large quantity. Indeed, the authors observe significant propofol levels in the blood plasma (in the jugular vein) immediately following FUS treatment of frontal cortex^55^, which likely has nonspecific effects. Such systemic plasma levels are nearly half of the systemic propofol levels necessary to observe anesthetic effects^57^. Interestingly, this method of FUS-mediated drug delivery and inhibition also shows highly transient effects (that lasts 8–14 s), reminiscent of the transient effects observed with FUS alone (without nanoparticles)^58^. It would also be interesting to know whether FUS may be enhancing (albeit nonspecifically) the freely circulating propofol’s focal delivery or may be working synergistically. However, perhaps the greatest future challenge in the use of nanoemulsions is that unlike the gas-filled microbubbles, they cannot be spatially concentrated due to their significantly lower responsiveness to acoustic radiation forces, making further improvements in their efficacy and specificity more challenging. Since our UC-carriers require orders of magnitude less drug than that required for systemic delivery to yield significant response, even if the payload is to be completely released systemically, it causes no detectable off-target effects (Figs. 3c, d and 4).

Focal delivery efficacy of AU-FUS with respect to off-target release is always better than that achievable by prolonged exposure to any uncaging (fragmentation) FUS sequence alone. First, the longer the exposure, the more drug gets released into the circulation due to the limited systemic lifetime of drug carriers, increasing off-target effects we want to avoid in the first place. Second, due to higher radiation pressures, prolonging the uncaging FUS sequences can both cause tissue damage^59^ and also push the microbubbles away from the focal target. Indeed we observe a reduction of the focally deposited drug in the presence of uncaging FUS sequence alone (Supplementary Fig. 1f), which has also been reported by Ferrara and colleagues^29^. To avoid this, the uncaging sequences should be brief and temporally spaced out from each other, leaving large temporal gaps where no uncaging occurs. Subsequently, the only mechanism to increase the focal efficacy of drug release is to introduce aggregation sequences in between the uncaging sequences to capture and make more of the systemic drug carriers available locally for subsequent uncaging.

Existing clinical ultrasound systems for targeting brain are expensive and bulky, which would make chronic FUS treatments challenging. In addition, although sub-MHz frequencies are used clinically to reduce the distortion and dissipation of ultrasound waves through the human skull, we chose to optimize our method for 2.5 MHz to achieve higher spatial resolution to target smaller brain areas in animal models. Yet, the use of miniaturized Microelectromechanical Systems (MEMS)-based ultrasound transducers^60^ can address both challenges for clinical translation at once: such microdevices are capable of generating sufficient ultrasound powers and can also be chronically implanted beneath/within the skull, potentially allowing the use of higher frequency ultrasound waves (e.g., 2.5 MHz) with higher spatial resolution in clinical applications. Another solution to reduce frequent dependency on large FUS systems can be to use drugs with long lasting effects such as the NMDAR antagonist ketamine (recently approved as a rapid-onset antidepressant for patients with treatment resistant depression), which is efficacious for weeks following acute administration^61^. Targeted delivery of ketamine could reduce its side effects (such as psychotomimetic and perceptual disturbances, in addition to heart rate and blood pressure complications^62^), thereby significantly enhancing its therapeutic index.

While our mechanistic understanding of psychiatric and neurological disorders has advanced in the preceding decades, translation of our knowledge or testing of hypotheses towards viable treatments has been extraordinarily challenging. This is in large part due to the vast complexity and heterogeneity of the brain. The ability to target drugs specifically to the pathological brain regions may offer a strategic path towards treatment of many CNS disorders. Such targeted delivery may also offer a new avenue for small molecules that have failed due to toxicity or lack of efficacy, by allowing drugs to be delivered only to the desired brain areas and possibly at higher concentrations than achievable through systemic delivery. Approved drugs should also benefit from focal delivery as this can likely eliminate, many if not all, side effects. Since individual chemical and ultrasound components of our methodology are already FDA approved, it might enable rapid repurposing of many existing or late-stage drugs.

## Methods

### Ultrasound-controlled drug carriers (UC-carriers)

We created ultrasound-controlled-drug carriers as shown schematically in Fig. 2a. The UC-carriers contained a backbone of 1,2-distearoyl-sn-glycero-3-phosphocholine (DSPC) (Avanti Polar Lipids) and 1,2-distearoyl-sn-glycero-3-phosphoethanolamine (DSPE)-polyethylene glycol (PEG)2000 (Corden Pharma) in 90:5 molar ratio. The remaining 5% was DSPE-PEG5000-Thiol (SH) for liposomes and DSPE-PEG5000-Maleimide (MAL) (both from Nanocs) for the bubbles. The lipids were dissolved in chloroform and mixed in appropriate volumes to achieve a total concentration of 2 mg mL^−1^ for the bubbles and 10 mg mL^−1^ for liposomes. Chloroform was then evaporated under nitrogen and kept overnight under vacuum. The resultant lipid films were stored at −20 °C till further use. For the bubbles, the lipid films were rehydrated with 1x phosphate-buffered saline (PBS) containing 10% propylene glycol and 10% glycerol. The solution was then heated at 70 °C for at least 30 min, and bath sonicated for at least 20 min or until the solution was clear. The headspace in the vial was filled with perfluorobutane (PFB; SynQuest Laboratories), and microbubbles were formed through a probe tip sonicator (70% power; Branson SLPe with 3 mm tip). The microbubble solution was then size isolated by centrifugation at 300 × *g* for 3 min, three times. After each centrifugation the wash solution was discarded and the remaining bubbles were resuspended in PBS:EDTA (1 mM EDTA; pH 6.5). The liposome lipid films were rehydrated with 1x PBS, heated at 70 °C for at least 30 min, and bath sonicated for 3 h. Drug (muscimol from Hellobio, sodium fluorescein from Sigma-Aldrich) was added at a drug/lipid ratio of 0.3 and 15 freeze-thaw cycles were performed in liquid nitrogen and 37 °C water bath, for 2.5 min each (all steps performed in the dark for fluorescein). The liposomes yielded a mean size of ~116 nm (Supplementary Fig. 3). The resultant bubbles and liposomes were mixed and allowed to conjugate overnight at 4 °C. Next day the solution was washed two times by centrifugation at 300 × *g* for 3 min. The concentration and size distribution were analyzed in triplicate using Multisizer 4 (Beckman Coulter). The mean size of the UC-carriers was ~1.7 μm (Supplementary Fig. 3).

### Animal preparation

Female Long Evans Rats (200–300 g, Charles Rivers Laboratories, Research Models and Service, Germany and Janvier Labs, Rodent Research Models and Associated Services, France) were used. The animals were housed in groups in standard IVC cages (Allentown), and had ad libitum access to food and water, and were on an inverted light cycle (12 h dark/12 h light). All procedures were approved by the Veterinary Office, Canton Zürich, Switzerland.

### Surgery

Rats were anesthetized in an induction chamber with 4–5% oxygenated isoflurane for 3–4 min. They were then moved to a preparation area where the tail vein was catheterized with a winged 27 G catheter (Terumo), and the head was shaved. In all, 2 mg kg^−1^ Meloxicam (Metacam) and 7 mL kg^−1^ warmed Lactated Ringers solution (Fresenius Kabi, AG) were subcutaneously injected every 3–4 h. The rat was moved to a stereotaxic frame (David Kopf), and an incision was performed on scalp to expose skull surface. A layer of eye cream was put on the eyes. A craniotomy was performed with a micro-drill above vM1 (Coordinates AP: 0–2.5 mm and ML: 0–2 mm, with respect to bregma), and dura was carefully opened with a 30G needle. During the craniotomy, the skull was frequently flushed with Ringers solution (B. Braun) to prevent heating. After dura removal, a piece of gel foam (Pfizer) was put on brain and Ringers solution was regularly applied to keep the brain moisturized until electrode insertion.

### Whisker stimulation

Whiskers were cut to around 15 mm length. The 8–12 thickest whiskers were inserted into a glass capillary tube, which was attached to a piezo actuator (T223-H4CL-503X, Piezo Systems). The piezo actuator was shielded with a custom-made copper cover and positioned with the help of a FISSO arm (S-20, Baitella, Switzerland). The whiskers were deflected with 120 Hz cosine pulses (292 mm s^−1^ velocity, displaced 2.34 mm in 8 ms), which were generated in LabView (National Instruments) and converted into an analog signal (DAC NI, USB-6211) and then drove the piezo actuator, adapted from Musall et al.^63^. Stimulus presentation was synchronized with the electrophysiological recordings with a TTL signal at stimulus onset. Whiskers were continuously stimulated at a repetition rate of 0.3 Hz.

### FUS setup and transducer calibration

A custom-made transducer (Sonic Concepts) with 2.5 MHz center frequency, 40 mm diameter, 30 mm working distance (20.65 mm focal depth), and  with 0.5 × 0.5 × 2.5 mm theoretical focal volume (−6 dB) was used, along with a custom impedance matching network for the transducer (Sonic Concepts). Calibration was done in a degassed Milli-Q water filled chamber with a 0.2 mm needle hydrophone (Acoustic Precision). The driving pulses for the transducer were produced by a function generator (Agilent 33210A, Keysight technologies) and controlled by a custom MATLAB script. A PicoScope (3205B) is used to control the pulse-repetition frequency (PRF) of the driving pulses. The signal was amplified 50 dB through a power amplifier (E&I 325LA).

### Electrophysiology and FUS drug delivery for vS1-vM1 measurements

All electrophysiological data were recorded with a RHD2000 system (Intan Technologies) with 30 kS s^−1^ sampling rate. All stereotaxic coordinates were determined with respect to bregma. A 32 channel Neuronexus probe (A2x16-10mm-100-500-177-A32, 15 or 50 μm thick) attached to a motorized three-dimensional (3D) arm (StereoDrive-960HD, Neurostar), fixed outside of the stereotaxic frame, was inserted into the vM1 (Coordinates AP: 1–2 mm, ML: 0.5–1 mm, depending on the vasculature, DV: 1.5–2 mm from pia, TZ region^33^) at 50° to the coronal plane (see Fig. 3a). The probe was initially inserted ∼250–500 μm below the cortical surface. The FUS transducer was integrated with an acoustic collimator, which was filled with degassed Milli-Q water and contained a polystyrene film (McMaster-Carr) at the end. The collimator’s shape was designed according to the FUS transducer’s geometry so that it would not interfere with the FUS beam. This assembly was stereotaxically positioned such that the FUS focal volume was targeted to vS1 (Coordinates AP: −2.3 mm, ML: 6 mm, DV: 3.3 mm from skull surface) at a 30° angle with respect to the sagittal plane such that the focal volume of FUS beam targeted cortical layers of vS1. A sufficient amount of warmed (to 37 °C) sterile ultrasound gel (Parker Laboratories) was put on the skull over vS1 for acoustic coupling. After positioning the FUS transducer, the recording probe was further inserted below cortical surface to reach a final DV position of 1.5–2 mm (tip). Following this, there was a period of about 1–2 h during which the wEP amplitudes stabilized. Baseline wEP responses were acquired for 10 min, followed by the intravenous injection of small-molecule- or vehicle-loaded UC-carriers (2−2.5 × 10^9^ total UC-carriers per animal) and/or muscimol (250 ng, Fig. 3c, d) injection intravenously with an injector (Genie Touch Syringe Pump, Kent Scientific) at a speed of 0.2 mL min^−1^. Thirty seconds after the start of injection, FUS sonication was done for 25–30 min (period of IV drug delivery). Electrophysiological data were recorded until at least 1 h after the end of sonication to see the complete drug effect and recovery. Immediately before (Supplementary Figs. 7 and 12) or within half an hour of sonication (Fig. 5 and Supplementary Figs. 2 and 8), the animals were injected IV with 1 mL of 0.5% Evans Blue (EB) dye to check for BBB integrity. EB dye was allowed to circulate for 30 min (Fig. 5, Supplementary Figs. 2 and 12) to 2 h (Supplementary Figs. 7 and 8) before transcardial perfusion and brain excision. Isoflurane was kept around 2.5–3% during all surgical procedures. During electrophysiological recordings and drug delivery it was maintained at around 1.5–2% to keep the anesthesia minimally low throughout the experiment. The anesthesia was regularly monitored visually with breathing rate and spontaneous LFP activity.

### Electrophysiology and FUS drug delivery for vS1-V1 measurements

The protocol used for vS1-V1 was the same as vS1-vM1 paradigm with the following exceptions: FUS sonication coordinates were changed to AP: −2.3 mm, ML: 6.5 mm, DV: 3.3 mm from skull surface and FUS angle was changed from 30° to 34° to create space for electrode insertion to V1. A more lateral region of vS1 was sonicated to achieve better coupling with skull with the new angling of the FUS transducer. Electrode insertion coordinates were changed for V1 recording to AP: −5.5 to 6.0 mm, ML: 3.2–3.5 mm, DV: 1.6–2.0 mm to ensure that recording is confined to cortical layers of V1. The angle of the probe insertion was kept at 50° as in vM1 recording. Instead of wEPs, VEPs were recorded by visual stimulation. The amount of muscimol-loaded UC-carriers per animal was changed from 2–2.5 × 10^9^ to 2.5–3.0 × 10^9^ and sonication period was changed from 25 to 30 to 30–35 min to show that even excessive muscimol delivery does not diffuse off-target brain circuits.

### Visual stimulation

A thin layer of eye cream was put on the contralateral eye. A 5 mm green LED was inserted in custom-made black rubber cone. The cone was positioned on the contralateral eye, with the help of a FISSO arm (XS-130, Baitella, Switzerland), such that the LED illuminates on the eye 3–4 mm away from the cornea. This configuration allowed sufficient intensity light stimulation to the eye, as the cone covered the eye and blocked any light from outside. The LED was triggered with a 10 ms TTL pulse, which was generated in LabView (National Instruments) and buffered through a NI-USB-6211 (National Instruments) board. Stimulus presentation was synchronized with the electrophysiological recordings with the same TTL signal used for stimulation. The eye was continuously stimulated at a repetition rate of 0.3 Hz. The ipsilateral eye was covered with a black rubber cone, with the help of another FISSO arm (XS-130, Baitella, Switzerland), after putting sufficient amount of eye cream. The whole recording session was done while all the lights in the room were turned off.

### Simultaneous vS1 and vM1 wEP recordings

Animals were prepared for craniotomy as indicated above and stereotaxic coordinates were determined with respect to bregma. An incision was made on the scalp and craniotomies were performed on vS1 (Coordinates AP: –2.3 mm, ML: 6 mm, DV: 1.1 mm from pia) then on vM1 (Coordinates AP: 1.5 mm, ML: 1 mm, DV: 0.8 mm from pia). vS1 was covered with gel foam, and continuously supplied with Ringers solution to keep the brain fresh until the vM1 craniotomy was completed. Eight to 12 whiskers were inserted into a capillary tube attached to a piezo stimulator. The first probe was inserted to vS1 with a 30° angle to the sagittal plane, 5 min later the second electrode was inserted to vM1 parallel to the sagittal plane. Neuronexus, A4x8-5mm-100-200-177 probes were used for both recordings in the experiment. Once both probes were inserted, whiskers were continuously stimulated at 1 Hz, wEPs were allowed 1–2 h to stabilize and responses were subsequently recorded for 8 min.

### In vitro FUS characterization

UC-carriers (5 × 10^8^ MB mL^−1^) flowed through a porous (13 kDa pore-size, 200 μm ID) microdialysis tube (132294, Spectra/Por), which was surrounded by agarose gel (0.6% in Milli-Q water + 0.9% NaCl, 16500500, UltraPure Invitrogen) in a custom-built channel. The custom-built channel that held the agarose gel had five marked sites, each of which was sequentially brought in the confocal alignment of a water-immersion objective (CFI APO NIR 60X W, Nikon), and the FUS transducer in a water tank containing degassed Milli-Q water. The first four of the five sites in direction of flow were sonicated with the test FUS pulse sequences and the last site served as a control site. For either standard-FUS or AU-FUS characterizations, each site was sonicated for the same amount of time (5 min), similar to Shortencarier et al.^29^ The tubing was then retracted from the agarose gel and each of the five agarose-gel sites were cut out and melted in heated (80 °C) deionized water, and fluorescence was measured with a plate reader (the control site was subtracted from all readings) (Gen5 Microplate Reader, BioTek). The reference measurement for normalization was fluorescein-loaded UC-carriers.

### Three-dimensional (3D) scanning of skull effects on FUS

Rat skulls were extracted, and tissue was removed from the surface before degassing in a chamber for 30 min to remove any trapped air inside the skull cap. The skulls were then positioned inside a degassed Milli-Q water chamber and controlled manually by mounting them on a 3D stage (PT3/M, Thorlabs). The water chamber was at the base of a stereotaxic system (Neurostar). A metal pointer was mounted on the motorized arm of the stereotaxic system to find bregma. The FUS transducer subsequently replaced the metal pointer on the stereotaxic arm such that the focal point matches precisely with tip of metal pointer. The FUS transducer was then positioned such that the focus of the ultrasound was aligned at 4 mm rostral to bregma and 7 mm ventral from skull surface. A needle hydrophone (9 μm thick gold electrode PVDF film; 0.2 mm tip size, Precision Acoustics) was mounted on a motorized stage (PT3/M-Z8), and then moved to find focal point of the transducer. The hydrophone scanned an area (4 × 6  × 5 mm) in 3D with skull and (4 × 6 × 4 mm) without skull. The motors were moved with 100 μm step sizes to scan the AP and DV axes, and ML axis moved continuously to decrease time required for scanning, while data was collected with 14 μm resolution. ML values were then sampled every 100 μm. An optical displacement sensor (SICK, OD Mini) was used to find the position of ML motor, and an analog-to-digital-converter (ADC) (NI-6009) was used to gather sensor data to the computer. Hydrophone pressure readings were collected with a PicoScope (3205B), and the entire setup was controlled with a custom MATLAB script. See Supplementary Fig. 4 for setup.

### Calculation of skull transmission factor

To measure the transmission factor of the skull, the peak-negative pressure was measured with the hydrophone at the focal point of the transducer (P1), following which the skull was moved in between. As the skull acts as a lens, the focal area changes slightly (depending on skull region and thickness). Hence, the hydrophone was repositioned to find the new focal area and peak-negative pressure was measured again (P2). The transmission factor is calculated as (P2 P1^−1^) × 100. For the brain region 4 mm rostral and 7 mm ventral to bregma, the transmission factor was 0.43 (57% loss); however, for skull region above vS1 we expect ~70% loss due to greater skull thickness. Our data is in agreement with previous findings^64^. See Supplementary Fig. 5.

### Electrophysiology data analysis

All electrophysiological data analysis was done in Python, version 3.6, using custom scripts. For evoked potential (wEP→vM1 and VEP→V1) analysis, the raw data was low-pass filtered (3rd order Butterworth filter) at 300 Hz. The wEPs and VEPs were extracted based on the time stamp of the whisker/visual stimulus. The waveforms were then corrected for amplitude offset by taking the mean of a 25 ms time-window preceding the stimulus onset. The peak-negative value for the wEP and VEP was considered to be the amplitude of the wEP and VEP response and used for analysis and data visualization. The four recording sites (i.e., electrodes) with the highest response amplitudes were then automatically selected for each experiment. Extracellular spike detection and sorting was done with Klustakwik, an open source software^65^. PSTHs were then extracted through a custom code in Python. For wEP and VEP analysis, moving averages were calculated for a window step size of 180 whisker deflections/visual stimuli (moving step size is 1 deflection/visual stimulus). Responses were normalized for each electrode to the average response of the 10 min window preceding FUS. For statistical analysis, in order to keep the number of data points for baseline (10 min, 152 peaks) and post treatment (30 min, 457 peaks) same, we randomly selected 152 peak values from post treatment. Data was visualized using Prism 7.0 and 8.0 (GraphPad).

### Measurement of brain temperature at the sonication site

The temperature probe (0.4 mm diameter, IT-21, Harvard Apparatus) was inserted through a 21G metal needle such that the tip of the probe stayed in the open cavity of the needle at the tip. This diameter (0.4 mm) was selected because it is smaller than the ultrasound wavelength (0.62 mm). A small craniotomy was performed on the skull in the following coordinates of AP: –2.3 mm, ML: 2–2.5 mm and the probe was inserted at a 52° angle. The FUS traducer was positioned with a collimator and coupling gel on vS1 at 34°. Brain temperature was allowed to stabilize for 1–2 h after probe insertion. Temperature recording began 10 min before vehicle-loaded UC-carriers (2.5 × 10^9^ total UC-carriers per animal) were intravenously injected at a speed of 0.2 mL min^−1^. Thirty seconds after the start of injection, FUS sonication began and continued for 30 min (period of UC-carrier injection), and temperature was continuously recorded until 10 min after sonication finished. The probe was connected to a portable thermocouple thermometer with 0.1 °C resolution (Harvard Apparatus). The output of the thermocouple was digitized through an Analog-to-Digital Converter (ADC) (NI-6009, National Instruments). The corresponding output voltage from the ADC was converted to temperature and further analyzed using a custom MATLAB (MathWorks) script based on the probe’s calibration sheet from the manufacturer. The data was collected at 10 Hz, a moving average was applied (window size of 10 s and step size of 0.1 s), and then further down sampled to 1 Hz for analysis. The data was visualized in Prism 8 (GraphPad).

### IVIS spectrum imaging

At the end of the experiment, animals were anesthetized with ketamine (100 mg kg^−1^) and xylazine (10 mg kg^−1^) prior to transcardial perfusion. Blood was cleared with PBS solution and animals were perfused with 4% paraformaldehyde solution (PFA; in PBS at pH 7). Brains were removed and placed in 4% PFA for at least 72 h before sectioning with a compresstome. Sections were cut at 100 μm thickness into a PBS bath and mounted onto microscope slides in Milli-Q water. Sections were dried in the dark and subsequently imaged using the IVIS Spectrum (Living Image). The following parameters were used: Epi-Illumination, FOV: 6.6, FSTOP: 2, Binning: (M) 8, Exposure time: 1 s, Excitation: 465 nm, Emission: 680 nm, and scales are presented as radiant efficiency. Slides were imaged from ~bregma –2.28 ± 0.7 mm (AP) and ROI analysis was employed using dimensions slightly larger than the theoretical FUS focal volume (3.5 mm DV × 1.5 mm ML oval angled at 30°; also see Supplementary Fig. 7) at locations 6 mm (to top of ROI) from midline. Radiant efficiency values [(photons s^−1^ cm^−2^ sr^−1^) per (μW cm^−2^)] for ROIs in vS1 for regions ipsilateral and contralateral to FUS were measured and values were normalized to the mean value of the contralateral side.

### MRI imaging

Contrast-enhanced T1-weighted imaging was employed to visualize BBB disruption, similar to a previous study^66^. Two groups of animals were compared (AU-FUS vs. standard_3_-FUS, *n* = 3 each). After sonication animals were transferred into the MR scanner (Bruker 7T PharmaScan). Pre-Contrast T1-weighted images were acquired (TE/TR: 4.5 ms/146 ms; NEX = 3; FOV: 35  × 35 mm; matrix = 256 × 256; slice thickness: 0.5 mm; flip Angle = 82°). These images were repeated (post-contrast) after injecting a bolus of the MRI contrast-agent Gd-DTPA (Omniscan) at 0.3 mL kg^−1^. Additionally, TurboRARE anatomical images were acquired as a reference (TE/TR: 24 ms/4095 ms; NEX = 10; echo spacing factor = 8; rare factor = 8; slice thickness = 0.45 mm; matrix: 180 × 120; FOV 20 ×12 mm). The animals were kept at a constant anesthesia level of 2.5% and sacrificed at the end of the experiment. The signal enhancement analysis was done similar Kobus et al.^67^. A region of interest (ROI) (1 × 1 mm) was drawn around three adjacent post-contrast T1-weighted slices, based on the FUS reference stereotactic coordinates, while excluding ventricles from the ROI. The difference in pre- and post-sonication T1-weighted ROI mean values were calculated for ipsilateral and contralateral to FUS sites (Fig. 5 and Supplementary Fig. 9). The values for each group were pooled across animals, subsequently plotted and compared. In the case of Gd-DTPA injection before sonication, there was no pre-scan image and the total signal intensity was measured for ROIs ipsilateral and contralateral to FUS, and values were normalized to the mean value for the contralateral side (Supplementary Fig. 7).

### IgG staining

Rats were treated with either AU-FUS or standard_3_-FUS and remained under 2% isoflurane anesthesia for 3 h while maintaining body temperature with a thermometric heating blanket with rectal probe (rats were injected with Lactated Ringers and Meloxicam). Rats were injected with ketamine (100 mg kg^−1^) and xylazine (10 mg kg^−1^) prior to transcardial perfusion. Following perfusion, the brains were post-fixed for ~18 h before preparing sections with a compresstome (50 μm thickness). Sections were blocked with 0.3% H_2_O_2_ in PBS for 10 min at room temperature (RT), rinsed with PBS containing 0.25% triton X-100 (PBST), and blocked with PBST containing 10% normal goat serum. The tissue was then incubated with Biotinylated anti-rat IgG (Vector labs BA-9400) diluted 1:1500 in PBST overnight at 4 °C. Sections were rinsed three times with PBST, incubated in PBST containing horseradish peroxidase avidin D (diluted 1:8000; Vector labs A-2004) for 1 hr at RT, and washed three times with PBST before adding a solution containing 0.05% diaminobenzidine, 0.01% H_2_O_2_, and 0.3% imidazole in PBS for 10 min at RT. The sections were immediately rinsed three times and mounted on glass slides before applying Fluoroshield with DAPI and sealing cover slips.

### IgG image acquisition and analysis

All slides were imaged using Nikon Eclipse TI microscope with Ander Neo sCMOS camera (DC-152Q-C00-FI) and NIS Elements software (v14.13.04 64-bit). Brightfield images were acquired using 4x objective (Plan Fluor 4x/0.13) and LED 0.8% intensity with 10 ms exposure and 1 × 1 binning. Single-image tiles were acquired with 25% overlap, and automatic stitch blending, image registration, and shading correction was performed. All analysis was performed using FIJI. Three sets of three slices were analyzed, corresponding to the focal center (3 sections; ~ –2.3 mm relative to bregma) and the anterior and posterior ends of the focal volume (3 sections each; 700 μM posterior or anterior to the focal center). Oval shaped ROIs (3.5 × 1.5 mm, 30° angle) 6 mm medial to midline at the surface of the cortex were added and average pixel intensity was measured. As increased staining decreases mean intensity, the normalized intensities are presented as the ROI contralateral to FUS treatment over the ROI ipsilateral to treatment.

### PCD experiments and analysis

A single element FUS transducer (H-147, Sonic Concepts, USA) at 2.5 MHz frequency with −6 dB focal volume of 0.51 × 0.51 × 3.28 mm, 50 mm working distance (38.8 mm focal depth) was used. The transducer was calibrated with a 0.2 mm hydrophone (Acoustic Precision, UK) such that it generates pressures at the focal point identical to the custom-made transducer used for the drug delivery experiments. A broadband (10 kHz to 15 MHz) passive cavitation detector (PCD, Y-107, Sonic Concepts, USA) was confocally aligned with the focal area of the transducer through a 20 mm center hole of the transducer. The data was amplified with a 20 dB RF amplifier (Ramsey Electronics), and digitized with the PicoScope (5242D, Pico Technology, UK) at 15 bits resolution with 125 MS s^−1^ rate. See Supplementary Fig. 11 for PCD setup.

Rats were anesthetized with isoflurane, the head was shaved, and 2 mg kg^−1^ meloxicam and 2 mL of Lactated ringers were injected subcutaneously. Animals were then fixed on a stereotaxic setup (Kopf) and a midline incision was made to expose the skull for brain coordinates. A metal pointer was attached to a motorized arm of stereotaxic system to find bregma. Sterilized ultrasound gel was then applied on the skull for coupling. A water box with ultrasound transparent polystyrene film at the bottom was positioned on the skull and filled with degassed Milli-Q water. The ultrasound transducer was then attached to the motorized stereotaxic arm with a custom-made metal pointer such that its focal point precisely targets bregma. The transducer was then moved to the target brain regions with the motorized arm using Neurostar software. Three sites were sonicated on each animal with the following coordinates: AP = –4.67 mm, ML = –2.51 mm, DV = 2.3 mm (Fig. 6a), AP = –2.65 mm, ML = –2.03 mm, DV = 2.71 mm (Fig. 6b), AP = –2.65 mm, ML = + 2.03 mm, DV = 2.71 mm (Fig. 6c, d). For each sonication site 6 × 10^8^ UC-carriers were injected in 1 mL saline 60 s prior sonication. In all, 10 ms pulse and 1 Hz PRF was used for standard_3,4_ sequences with 0.75 MPa and 1.5 MPa pressures, respectively. The AU-FUS_in-vivo_ sequence was applied as described in the Table 1. Each sonication duration was 5 min.

The data was recorded with the PicoScope’s graphical user interface (PicoScope 6.14.10), and converted to MATLAB (.mat) format. For the standard_3,4_-FUS sequences, the first 2 ms from the PCD was used in the analysis. For the AU-FUS sequence, the data was analyzed from two different segments separately since the AU-FUS sequence consists of two distinct sequences; AU-FUS Aggregation and AU-FUS Uncaging sequences. As UC-carriers are expected to aggregate most strongly towards the end of the sequence, the last 2 ms of the AU-FUS Aggregation sequence was used for analysis. For the AU-FUS Uncaging sequence, since the first pulse sequence impinging on the aggregated bubbles is expected to give the strongest response, this pulse sequence (1000 cycles, 0.4 ms) is used for analysis. All the data was subjected to a Hamming window and plotted in the frequency domain after Fast Fourier Transform (FFT) in MATLAB 2015b.

Fifty kilohertz before and after the ultra-harmonics (3.75 and 6.25 MHz) was considered for area-under-the-curve (AUC) calculations. For standard_4_-FUS (1.5 MPa), the ultra-harmonics were detrended with a median filter (kernel size = 1000) to eliminate signal elevation due to the broadband emission background. For broadband emission calculations, 250 kHz before and after the integer and ultra-harmonics were excluded and the total range between 2.5 and 10 MHz was covered.

### Extraction of muscimol from loaded UC-carriers

Muscimol-loaded UC-carriers were prepared as stated above. After overnight conjugation of the microbubbles and liposomes, the solution was washed and centrifuged twice at 300 × *g* for 3 min to remove any unencapsulated muscimol in PBS:EDTA (1 mM EDTA; pH 6.5). The final microbubble cake was resuspended in PBS:EDTA (1 mM EDTA; pH 6.5) and the concentration and size distribution were analyzed in triplicates using Multisizer 4 (Beckman Coulter). The total volume was also noted. In all, 0.5 mL of absolute ethanol was added to the UC-carriers to dissolve the lipids and extract the muscimol. Following this, 2.5 mL running buffer (RB; 95% acetonitrile, 5% water, 0.1% formic acid) used in LC-HR-MS/MS detection was added. The solution was then bath sonicated at 70 °C. After this, 1.5 mL of the solution was centrifuged at 13,300 r.p.m. for 10 min, and the supernatant was aliquoted in triplicate (400 μL) and frozen at –80 °C until LC-HR-MS/MS detection. The dilutions were noted and factored in while calculating the total amount of muscimol.

### Quantification of muscimol with LC-HR-MS/MS

The stock solutions of muscimol (HelloBio) and internal standard (methanamine hydrochloride, Sigma-Aldrich, ISTD) were prepared in a mixture of acetonitrile/H_2_O 1:1 (v/v) (ULC-MS grade, Biosolve BV) at a concentration of 200 and 100 μg mL^−1^, respectively. The stock solutions were stored at 4 °C until use. The solutions used for the quantification calibration curve were prepared as a dilution series at the concentrations of 1000, 500, 250, 50, 10, 2, and 0.5 ng mL^−1^ in acetonitrile/H_2_O 1:1 (v/v) supplemented with ISTD at a final concentration of 200 ng mL^−1^. The effective weighted values are listed in Supplementary Table 1. The muscimol-loaded UC-carrier samples were diluted with acetonitrile/H_2_O 1:1 (v/v) by a factor of 1:50. A volume of 5 μL was injected for quantification.

LC-HR-MS/MS procedure was adapted from the method developed by Gonmori et al.^68^. Liquid chromatography was performed on an UltiMate 3000 UHPLC (Thermo Fisher, Waltham, MA, USA) build from a binary RS pump, an XRS open autosampler, a temperature-controllable RS column department and a diode array detector, all from the series Dionex UltiMate 3000. Compound separation was achieved at 25 °C on an ACQUITY UPLC Amide Column (100 Å, 1.7 μm, 2.1 × 100 mm; Waters, Milford, MA, USA). Eluent A consisted of H_2_O and eluent B was acetonitrile, both acidified with 0.1% formic acid (VWR International bvba). The following conditions were applied for elution at a constant flow rate of 0.3 mL: (i) linear decrease starting from 90% to 50% B during 3.5 min; (ii) switch to 10% B from 3.5 to 3.7 min (iii) holding 10% B until 7.0 min (iv) change until 7.2 min to the starting conditions of 90% B; (v) equilibration for 2.8 min until the next measurement run.

Mass spectrometry was conducted on a QExactive quadrupole-Orbitrap mass spectrometer (Thermo Fisher Scientific, Waltham, MA, USA) equipped with a heated ESI source operating under following conditions: needle voltage of 3.5 kV, sheath, auxiliary and sweep gas (N_2_) flow rates of 30, 15, and 0 (arbitrary units), respectively. The capillary and the auxiliary gas heater temperature amounted 280 and 250 °C, respectively. The data independent MS/MS mode (DIA) in positive ionization mode was selected including an inclusion list of the precursor ion corresponding to protonated molecules of muscimol (m/z 115.05020, 62 CE [collision energy], tR = 2.25–5.00 min) and ISTD (m/z 113.07094, 20 CE, tR = 0–2.25 min). A precursor ion isolation window of 3.0 m/z and a resolution of 70,000 at full width at half maximum (FWMH) were selected together with a maximum IT of 400 ms and an AGC target of 2 × 105. The ion chromatograms corresponding to the signals of the fragment ions of muscimol and the ISTD were extracted at m/z 98.02–98.03 and 96.04–96.05, respectively (see Supplementary Fig. 13). Xcalibur 4.1 and QuanBrowser 4.1 (Thermo Fisher Scientific) software were employed for data acquisition, and for peak-area integration and quantitation, respectively.

The recovery was estimated by first adding the standard solution containing 4.86 ng mL^−1^ muscimol in a 50x diluted sample containing 1.527 ng mL^−1^. A spiked concentration of 6.32 ng mL^−1^ was obtained (98.6% recovery). In the second standard addition, 19.44 ng mL^−1^ were added to another sample containing 1.589 ng mL^−1^. A spiked concentration of 22.212 ng mL^−1^ was obtained in this case (106.1% recovery).

Quantification was performed with the addition of the internal standard and the calibration curves were constructed by least-squares linear regression analysis. Thereby, peak-area ratios of the signals from the analyte and the internal standard were plotted against the concentration of the analyte. The set of calibrators at 0.5, 2, 10, 50, 250, 500, and 1000 ng mL^−1^ concentration were measured to determine the dynamic range and the linearity of the quantification method. The quadratic fitting and the weighting function of 1 × 2^−1^ were selected and correlation coefficient values *R*^2^ > 0.999 was obtained (see Supplementary Fig. 14). A deviation below 5% was obtained by comparing the weighted and the measured concentrations (see Supplementary Table 2).

### Statistical analysis

Non-parametric statistical tests (pairwise Man–Whitney rank-sum test, or Wilcoxon matched-pairs-signed rank test) were performed for electrophysiological data and imaging data. For PCD experiments one-tailed, unpaired *t*-test with Welch’s correction was performed. All statistical analysis was performed using GraphPad Prism version 7 and 8 for Mac, GraphPad Software, San Diego, California USA.

### Reporting summary

Further information on research design is available in the Nature Research Reporting Summary linked to this article.

## Supplementary information

Supplementary Information

Reporting Summary

Description of Additional Supplementary Files

Supplementary Movie 1

## Data Availability

Source data are provided with this paper. The data that support the findings of this study are preserved at repositories of Institute of Neuroinformatics, ETH Zurich and available from the corresponding authors upon reasonable request.

## Source data

Source Data

## Code availability

The code that support the findings of this study are available from the corresponding authors upon reasonable request.
