# Supplementary material for: Non-invasive molecularly-specific millimeter-resolution manipulation of brain circuits by ultrasound-mediated aggregation and uncaging of drug carriers
Source: Nat Commun. 2020 Oct 1;11:4929. doi: 10.1038/s41467-020-18059-7 (PMC7529901; doi:10.1038/s41467-020-18059-7)
Supplement: Supplementary file 1 — Supplementary Information [file 41467_2020_18059_MOESM1_ESM.pdf]

Supplementary information

Non-invasive molecularly-specific millimeter-resolution manipulation of brain circuits by ultrasound-mediated aggregation and uncaging of drug carriers

Ozdas, Shah, Johnson et al.

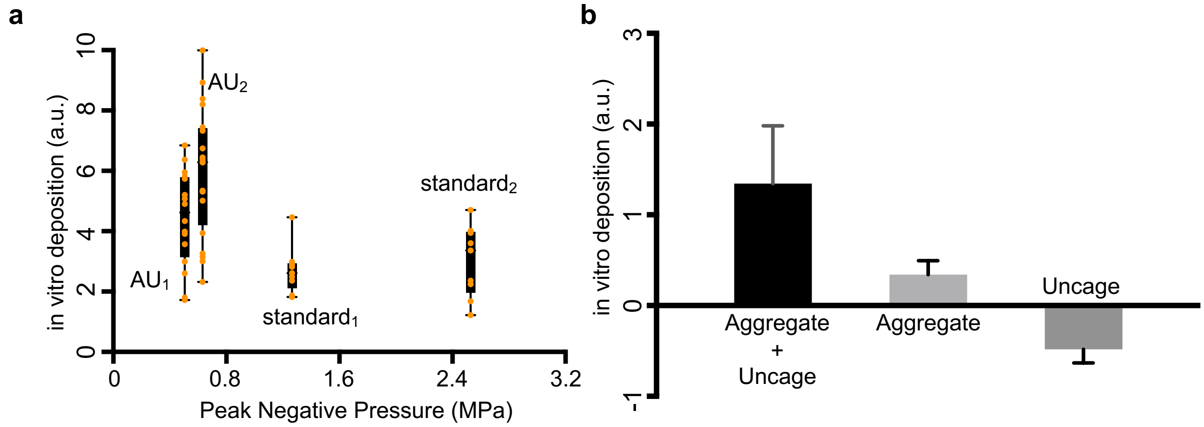

Supplementary Figure 1: In vitro assessment of drug deposition in microdialysis tubing using standard$_{1,2}$-FUS versus AU-FUS sequences

a Deposition of fluorescein released from UC-carriers in agarose around microdialysis tubing (normalized fluorescence; see Methods) after multi-component AU-FUS [AU$_1$-FUS (in vitro) (n = 16), AU$_2$-FUS (in vitro) (n = 20)] and single-component standard-FUS [standard$_1$-FUS (n = 9), standard$_2$-FUS (n = 9)]. See Table 1 for parameters. All data is represented as a box-and-whisker plot [min to max, showing all points (orange)].

b Deposition of fluorescein released from UC-carriers in agarose around microdialysis tubing (normalized fluorescence; see Methods) after AU$_3$-FUS (in vitro) sequence components. Both the radiation and uncaging pulses are required for efficient release (see parameters in Table 1). All data is mean ± s.e.m. (n = 16 for aggregate + uncage, n = 11 for aggregate only, n = 16 for uncage only). Note: These pressures are the same as AU-FUS$_{\text{in vivo}}$, accounting for skull attenuation.

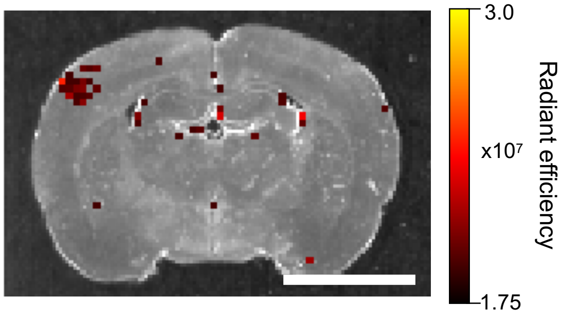

Supplementary Figure 2: In vitro optimized AU-FUS sequence causes BBB opening in vivo. IVIS spectrum imaging of Evans Blue dye extravasation following AU_1-FUS (in vitro) parameters optimized under in vitro conditions. BBB opening is observed in vS1 in the ipsilateral side to AU_1-FUS (in vitro) treatment. FUS parameters shown in Table 1 of main text ["AU_1-FUS (in vitro)"]; P_U was slightly lower (0.45 vs 0.5 MPa shown in Table 1). Scale bar is 0.5 cm.

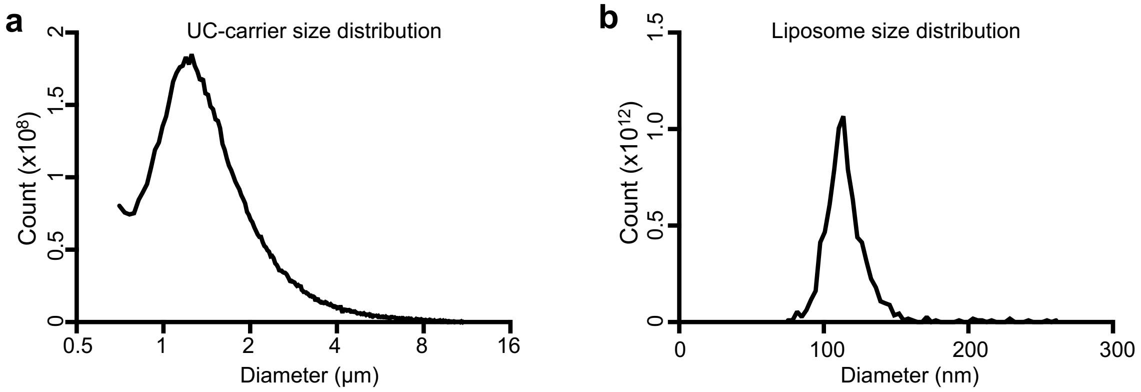

Supplementary Figure 3: Characterization of UC-carrier and liposome size distributions.
a Representative example of size distribution of UC-carriers with a mean diameter of 1.713 µm.
b Representative example of drug-loaded liposomes with mean diameter of 116 nm.

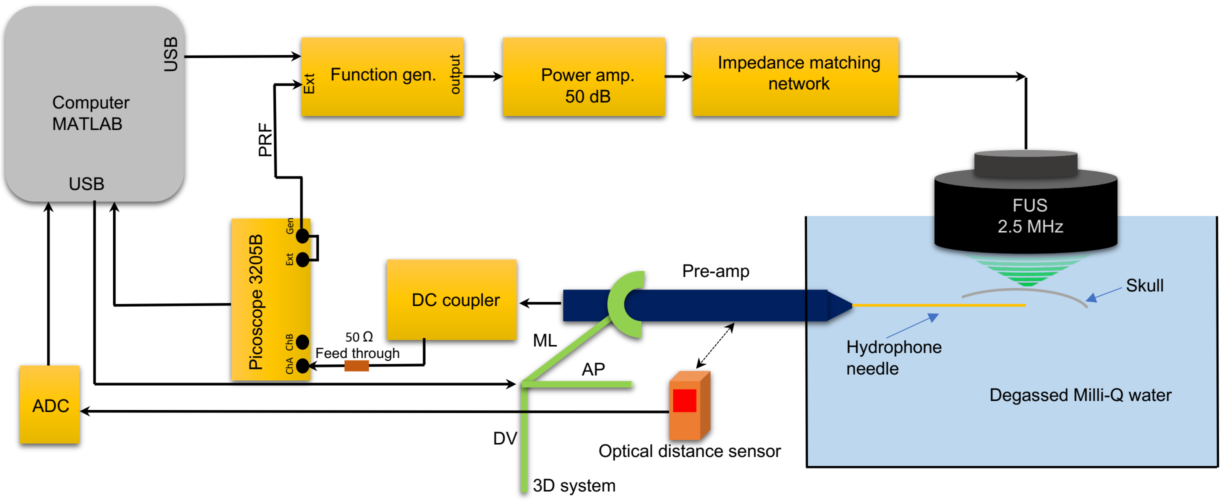

Supplementary Figure 4: Schematic illustration of system for automated pressure mapping to measure the effects of skull on FUS.
See Methods for further details.

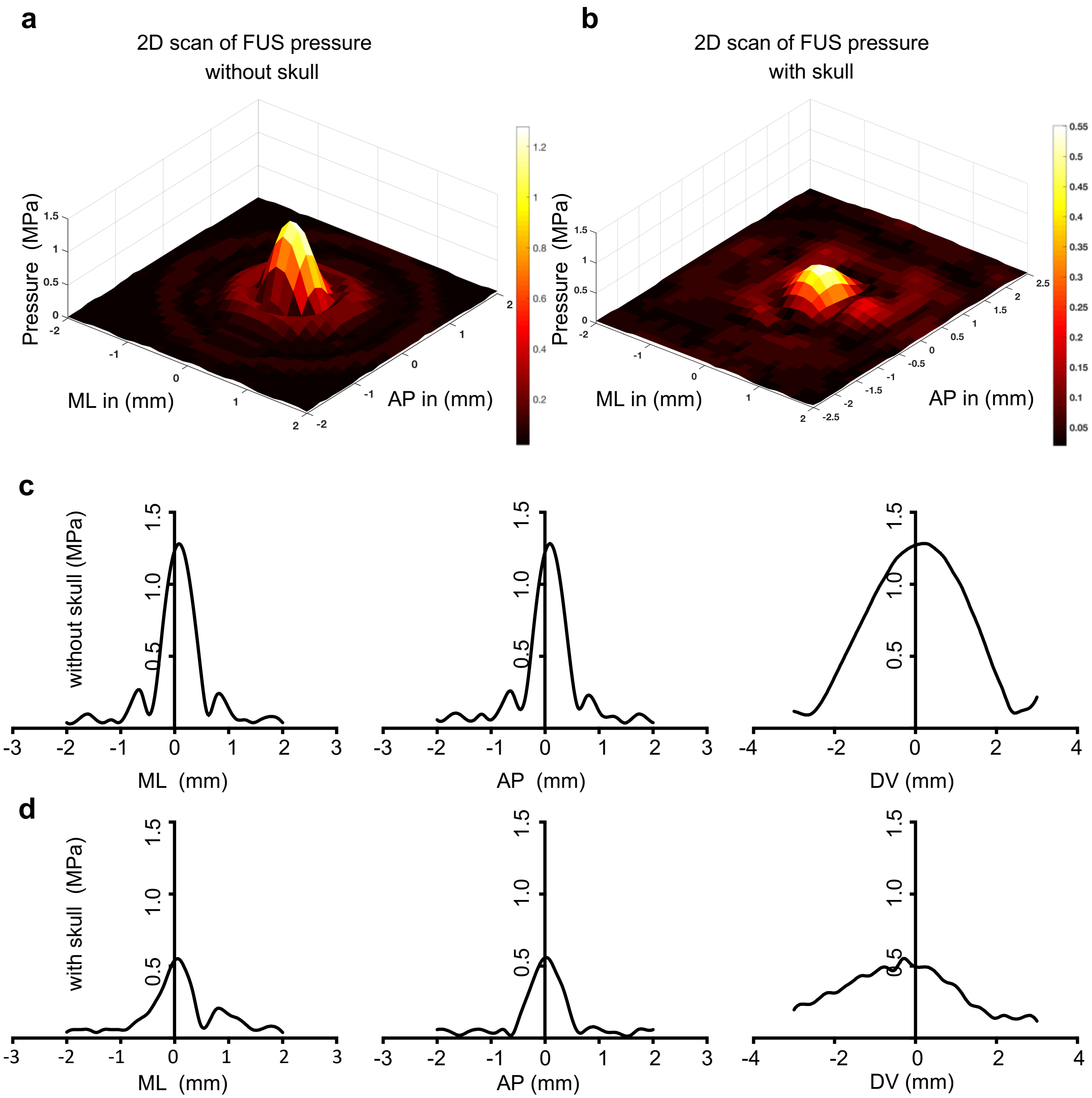

Supplementary Figure 5: 2D and 1D Scan of FUS waves with and without skull to determine the attenuation of FUS by the skull.
Intensity profile of the FUS transducer at focal plane: **a** without skull. **b** with skull.
FUS pressure intensity profiles along three axis (ML, AP, DV): **c** without skull. **d** with skull.

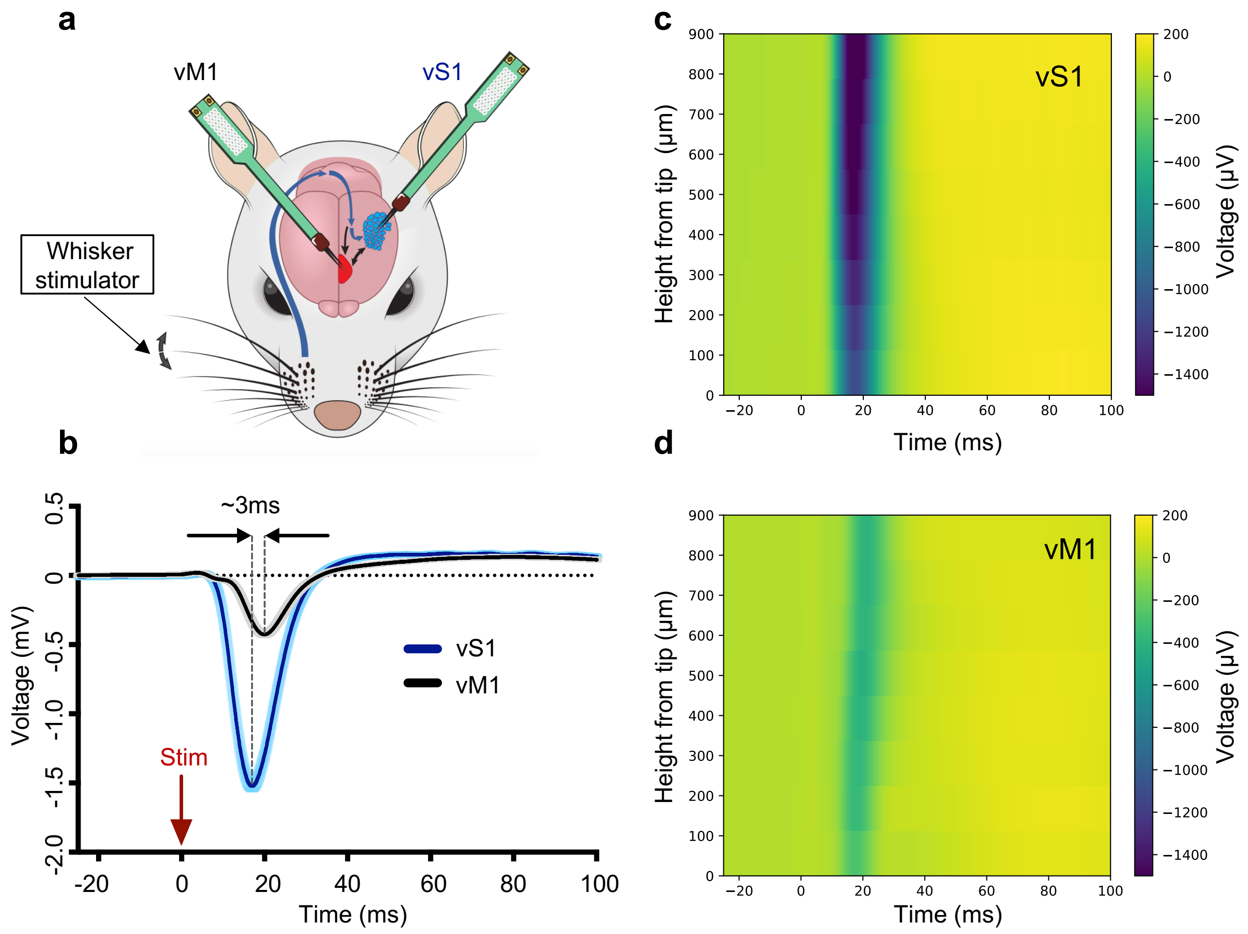

Supplementary Figure 6: wEPs recorded simultaneously from vS1 and vM1 following whisker stimulation shows directional propagation of neural activity from vS1 to vM1.

a Schematic illustration of experimental setup.

b Average of all wEPs (whisker deflection at 1 Hz for 8 mins, deflection at t = 0 ms) recorded simultaneously from vM1 (black) and vS1 (blue). Data shown is from strongest responding electrode in vM1 and vS1 in one animal. Note ~3 ms difference in latency between the peaks of wEPs from vS1 and vM1, consistent with previous data[49]. Data is mean ± s.e.m.

c Heatmap for one shank of the probe in vS1.

d Heatmap for one shank of the probe in vM1.

Heatmaps show the peak negative amplitude of the average of wEPs (µV) for all 8 electrodes (100 µm vertical spacing from tip-the most ventral electrode) in the probe shank following whisker deflection.

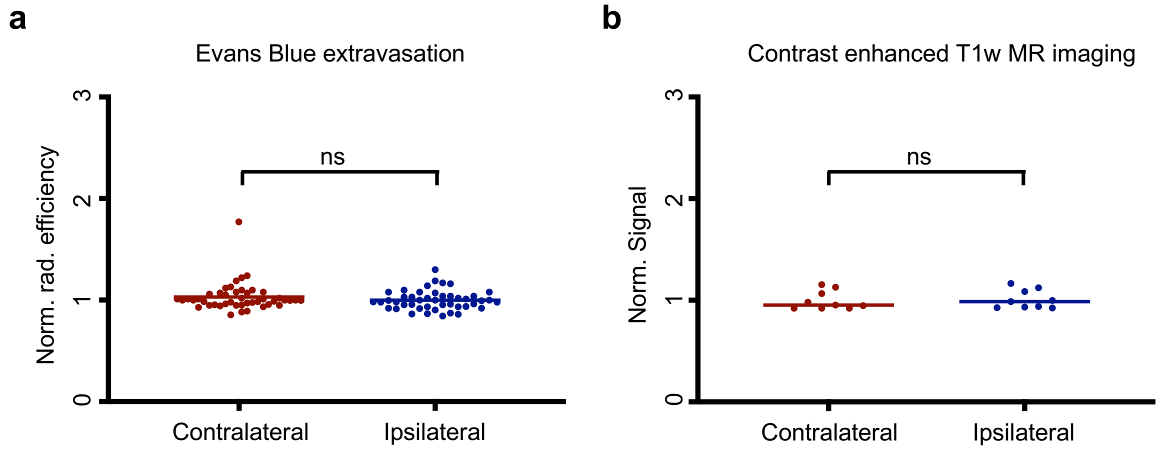

Supplementary Figure 7: No indication of BBB opening during sonication with AU-FUS as measured by Evans Blue or Gadolinium extravasation.

a Evans blue was injected immediately before sonication and allowed to circulate for 2-h post-sonication before transcardial perfusion. Regions of interest (ROIs) (1.5 mm x 3.5 mm, blue) were measured as radiant efficiency [(photons sec^-1 cm^-2 sr^-1) per (µW cm^-2)] ipsilateral to FUS application on vS1 and were compared to the contralateral vS1 on brain sections imaged with the IVIS spectrum. Radiant efficiency values within ROIs for AU-FUS were quantified [n = 45 (3 rats x 15 brain sections)]. Pairwise Mann-Whitney rank sum test AU-FUS (ipsilateral vs. contralateral, p= 0.3477).

b Animals were injected with Omniscan immediately before sonication and imaged after sonication. ROIs (1.0 mm x 1.0 mm, blue, approximate ROI location) were measured as signal enhanced T1-weighted MR images, following Gd administration, ipsilateral to FUS application, which were compared to the contralateral vS1. Ratio (ipsilateral to contralateral) of contrast enhanced T1-weighted MR image ROIs using AU-FUS were quantified [n = 9 (3 rats x 3 brain sections)]. Pairwise Mann-Whitney rank sum test, AU-FUS (ipsilateral vs. contralateral, p= 0.6048).

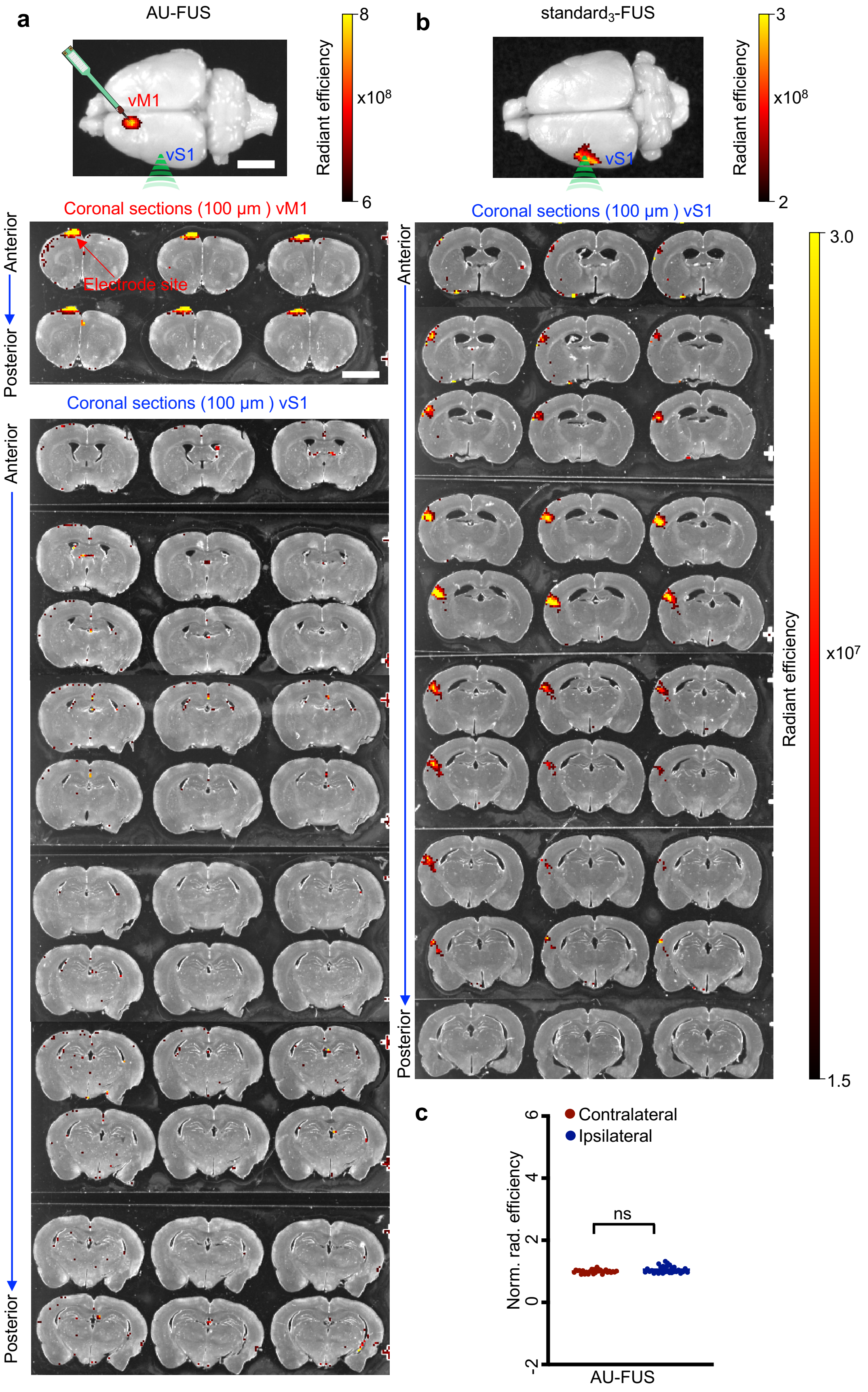

a
AU-FUS
vM1
vS1
Radiant efficiency
x10^8
8
6
Coronal sections (100 µm) vM1
Electrode site
Anterior
Posterior
Coronal sections (100 µm) vS1
b
standard3-FUS
Radiant efficiency
x10^8
3
2
Coronal sections (100 µm) vS1
3.0
x10^7
1.5
c
Contralateral
Ipsilateral
ns
Norm. rad. efficiency
AU-FUS

Supplementary Figure 8: Strong Evans Blue extravasation in the FUS focal volume after standard$_3$-FUS, but not AU-FUS treatment of vS1.

a Top: IVIS spectrum whole-brain imaging of a representative animal treated with AU-FUS and UC-carriers on vS1 (Note: The hot spot seen in the vM1 is due to electrode insertion). Evans Blue was allowed to circulate for 2 hrs before perfusion. Middle: Evans Blue extravasation in electrode insertion site in vM1 from the brain shown above. Bottom: Coronally sectioned IVIS spectrum images of focal area after AU-FUS treatment.

b Top: IVIS spectrum whole brain imaging of a representative animal treated with standard$_3$-FUS and UC-carriers on vS1. Bottom: Coronally sectioned IVIS spectrum images of focal area after standard$_3$-FUS treatment.

c Normalized radiant efficiency values within ROIs for AU-FUS [n = 30 (2 rats x 15 brain sections)]. Pairwise Mann-Whitney rank sum test AU-FUS (ipsilateral vs. contralateral, p = 0.1666).

Serial sections are arranged left → right and top → bottom (anterior → posterior).

Scale bar is 0.5 cm.

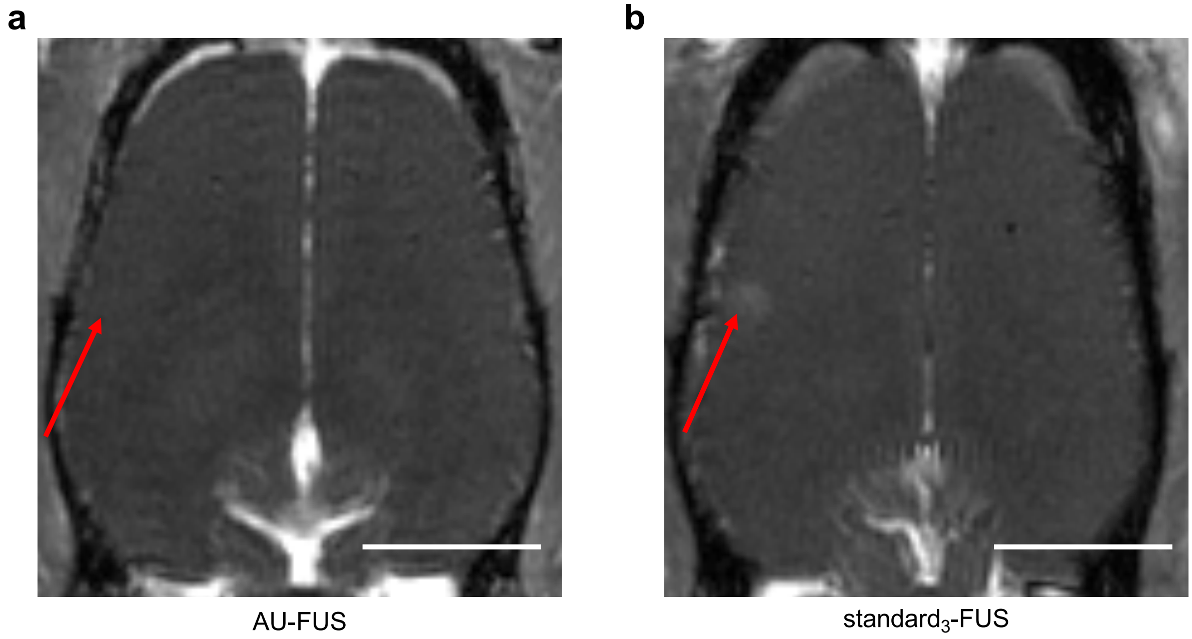

Supplementary Figure 9: Enlarged images from Fig. 5b showing contrast enhancement following standard$_3$-FUS, but not AU-FUS treatment.
a AU-FUS (left; red arrow)
b standard$_3$-FUS (right; red arrow).
Scale bar is 0.5 cm.

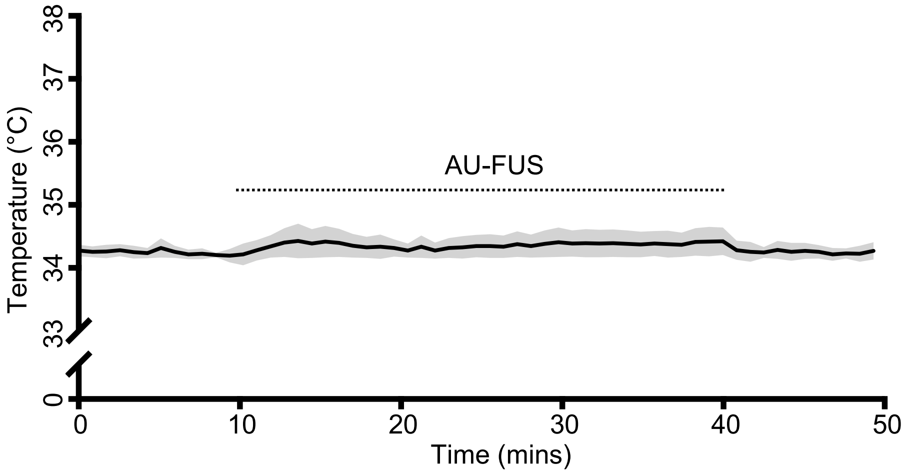

Supplementary Figure 10: Time course of local brain temperature during AU-FUS treatment with UC-carriers shows negligible temperature increase.

A thermocouple sensor was inserted at an angle below the skull to vS1 and the FUS transducer was positioned above. AU-FUS treatment was done through intact skull and temperature was monitored for baseline (0-10 mins), AU-FUS treatment (10-40 mins), and post-AU-FUS treatment periods (40-50 mins). The average change in temperature during AU-FUS treatment was negligible (0.12°C). All data is mean ± s.e.m. n = 3 rats.

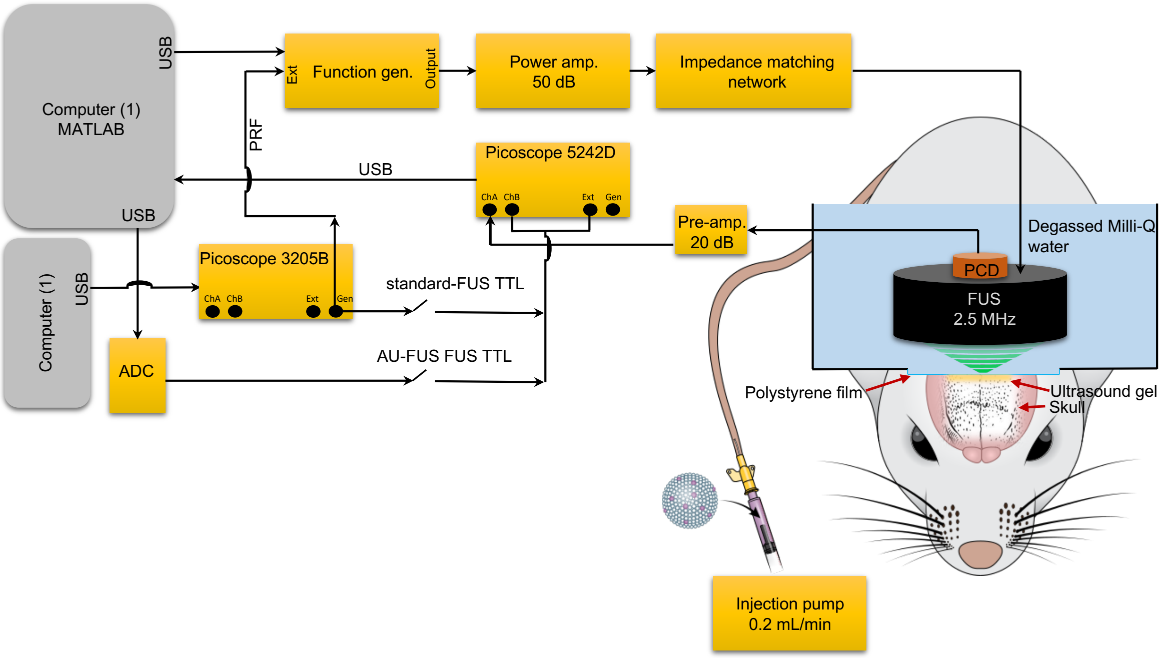

Supplementary Figure 11: Schematic illustration of system for passive cavitation detection of UC-carriers in vivo*.*
See Methods for further details.

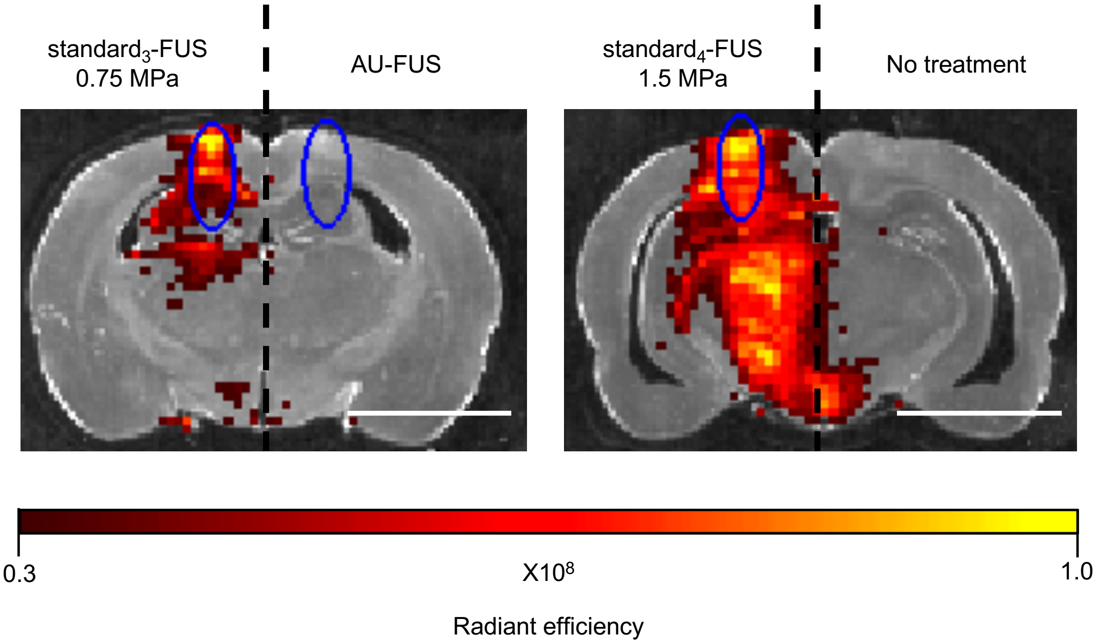

Supplementary Figure 12: BBB opening as predicted by stable and inertial cavitation signals from passive cavitation detection (PCD) measurements.
Evans blue extravasation is observed with standard$_3$-FUS (left hemisphere of brain section on the left; see FFT in Fig. 6b) and standard$_4$-FUS (left hemisphere of brain section on the right; see FFT in Fig. 6a), but not AU-FUS (right hemisphere of brain section on the left; see FFTs in Fig. 6c & d). Passive cavitation detection analysis indicated stable cavitation with standard$_3$-FUS and inertial cavitation with standard$_4$-FUS. Brain sections are from the PCD data presented from one animal in Fig. 6a-d. Blue ROIs indicate the focal area. Scale bar is 5 mm.

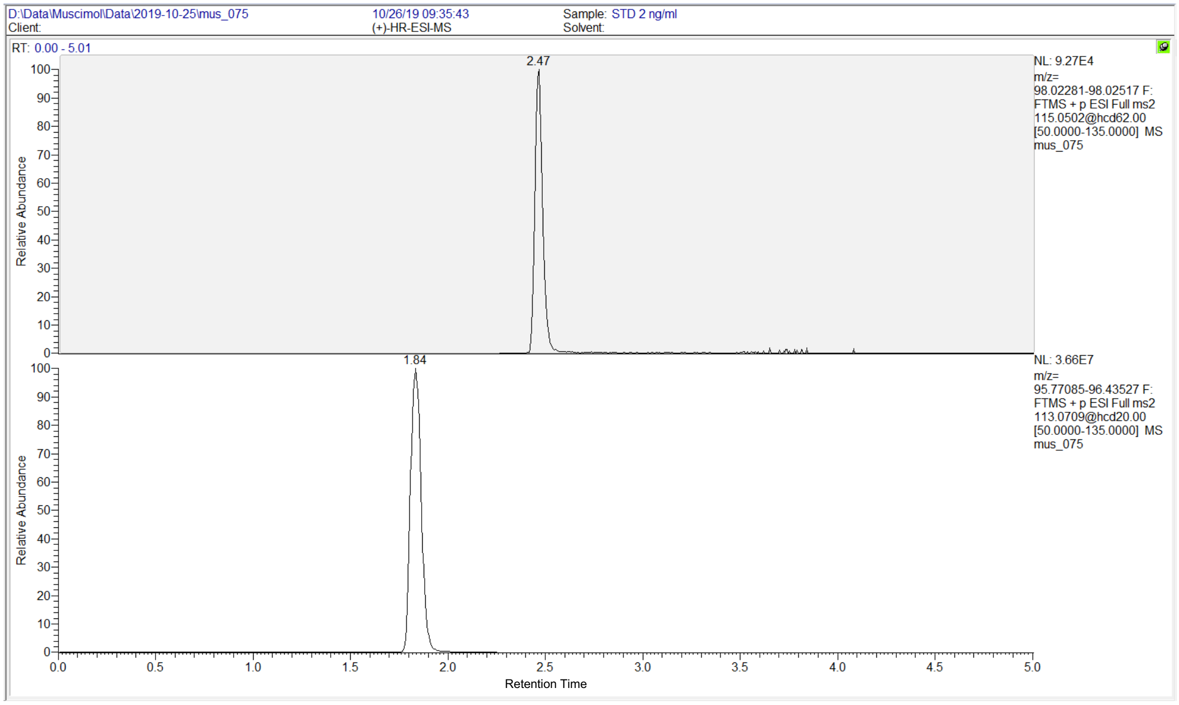

Supplementary Figure 13: Chromatograms of ISTD at 200 ng mL^-1 (top) and muscimol standard solution at 2 ng mL^-1 (bottom) concentration measured with LC-HR-MS/MS.

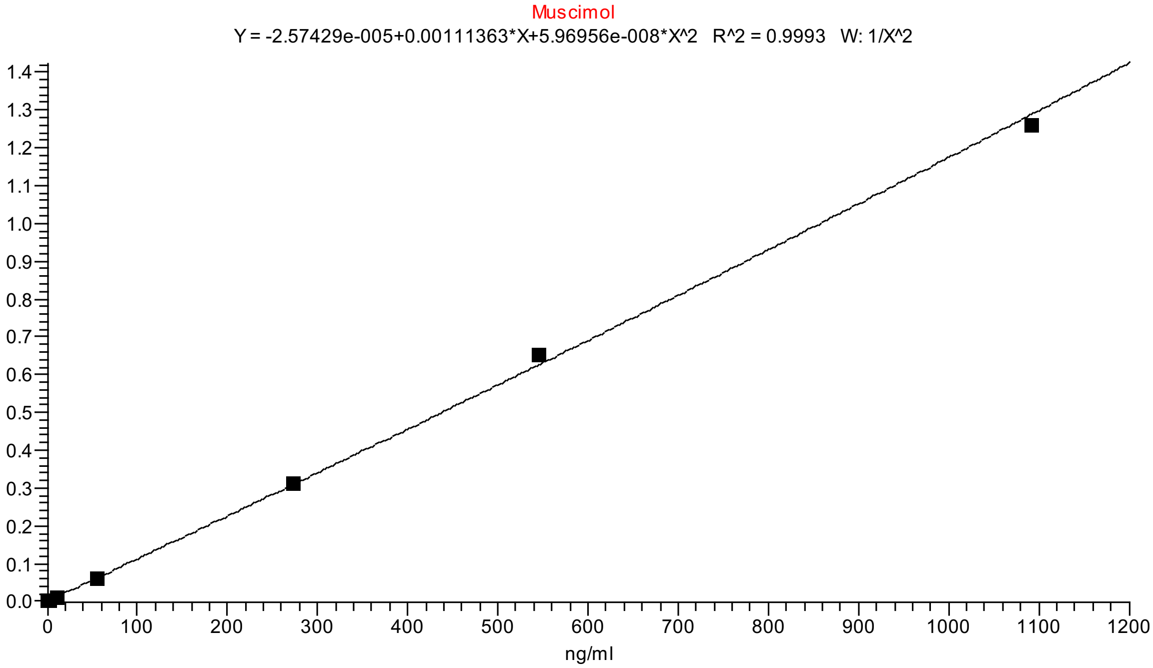

Supplementary Figure 14: Calibration curve of muscimol standard solutions used for quantification.

Supplementary Table 1

Preparation of the standard and ISTD solutions used for quantification.

**Initial weight**					
	Volume [mL]	Calc. [mg]	Eff. [mg]	Calc. [ng mL^-1]	Eff. [ng mL^-1]
Muscimol	10	2.00000	2.185	200'000	218'500
(3-methyl-1,2-oxazol-5-yl) methanamin ISTD	2	0.20000	0.232	100'000	116'000

**Muscimol**					
Solutions	Conc. calc. [ng mL^-1]	Conc. eff. [ng mL^-1]	Volume [mL]	Volume aliquot	
SL_(Muscimol)	200'000	218'500			
WS1_(Muscimol)	10'000	10'925	1	50 µL SL_(Muscimol)	
WS2_(Muscimol)	500	546	1	50 µL WS1_(Muscimol)	
WS3_(Muscimol)	25	27.3	1	50 µL WS2_(Muscimol)	
STD 1	0.50	0.546	1	20 µL WS3_(Muscimol)	
STD 2	2.00	2.185	1	80 µL WS3_(Muscimol)	
STD 3	10.00	10.925	1	20 µL WS2_(Muscimol)	
STD 4	50.00	54.625	1	100 µL WS2_(Muscimol)	
STD 5	250.00	273.125	1	25 µL WS1_(Muscimol)	
STD 6	500.00	546.250	1	50 µL WS1_(Muscimol)	
STD 7	1'000.00	1'092.500	1	100 µL WS1_(Muscimol)	

**ISTD**					
Solutions	Conc. calc. [ng mL^-1]	Conc. eff. [ng mL^-1]	Volume [mL]	Volume aliquot	
SL_(ISTD)	100'000	116'000			
WS1_(ISTD)	4'000	4'640.00	10	400 µL SL_(ISTD)	
STD 1	200.0	232.00	1	50 µL WS1_(ISTD)	
	200.0	232.00	3	150 µL WS1_(ISTD)	

Supplementary Table 2

Weighted and measured concentrations.

Weighted concentration [ng mL^-1]	Measured concentration [ng mL^-1]	% Diff
0.546	0.548	0%
2.19	2.16	-1%
10.9	10.6	-3%
54.6	54.9	1%
273	276	1%
546	569	4%
1093	1068	-2%
