# Supplementary material for: Non-invasive molecularly-specific millimeter-resolution manipulation of brain circuits by ultrasound-mediated aggregation and uncaging of drug carriers
Source: Nat Commun. 2020 Oct 1;11:4929. doi: 10.1038/s41467-020-18059-7 (PMC7529901; doi:10.1038/s41467-020-18059-7)
Supplement: Supplementary file 2 — Reporting Summary [file 41467_2020_18059_MOESM2_ESM.pdf]

nature research

Corresponding author(s): M.F. Yanik, A.S. Shah

Last updated by author(s): Jul 27, 2020

Reporting Summary

Nature Research wishes to improve the reproducibility of the work that we publish. This form provides structure for consistency and transparency in reporting. For further information on Nature Research policies, see Authors & Referees and the Editorial Policy Checklist .

Statistics

For all statistical analyses, confirm that the following items are present in the figure legend, table legend, main text, or Methods section.

n/a	Confirmed	
☐	☒	The exact sample size (*n*) for each experimental group/condition, given as a discrete number and unit of measurement
☐	☒	A statement on whether measurements were taken from distinct samples or whether the same sample was measured repeatedly
☐	☒	The statistical test(s) used AND whether they are one- or two-sided<br>*Only common tests should be described solely by name; describe more complex techniques in the Methods section.*
☒	☐	A description of all covariates tested
☒	☐	A description of any assumptions or corrections, such as tests of normality and adjustment for multiple comparisons
☐	☒	A full description of the statistical parameters including central tendency (e.g. means) or other basic estimates (e.g. regression coefficient) AND variation (e.g. standard deviation) or associated estimates of uncertainty (e.g. confidence intervals)
☐	☒	For null hypothesis testing, the test statistic (e.g. *F*, *t*, *r*) with confidence intervals, effect sizes, degrees of freedom and *P* value noted<br>*Give P values as exact values whenever suitable.*
☒	☐	For Bayesian analysis, information on the choice of priors and Markov chain Monte Carlo settings
☒	☐	For hierarchical and complex designs, identification of the appropriate level for tests and full reporting of outcomes
☒	☐	Estimates of effect sizes (e.g. Cohen's *d*, Pearson's *r*), indicating how they were calculated

Our web collection on statistics for biologists contains articles on many of the points above.

Software and code

Policy information about availability of computer code

Data collection	-Electrophysiological data was collected with Intan Recording System Software (2.6), we added additional custom functions to the software for real time LFP analysis to test drug effect.<br>-Skull data was collected with Matlab (2014a).<br>-In vitro data was collected with BioTek Gen5 v2.07<br>-MRI Image acquisition with Paravision 6, Bruker.<br>-IVIS spectrum data collected with Living Image software.<br>-IgG immunohistochemistry images were obtained using NIS Elements software (v14.13.04 64 bit).<br>-PCD data was collecting with PicoScope 5242D and Software version 6.14.10<br>-LC-HR-MS/MS acquisition was done using Xcalibur 4.1.<br>-Microbubble size and concentration was measured using Beckman Coulter Multisizer 4.01<br>-Liposome size and concentration was measured using Izon Control Suite V3.2.2.268
Data analysis	Electrophysiological data is analyzed with Python 3.6, skull data is analyzed with Matlab (2015b) and Prism 7 and 8.<br>-In vitro data and skull data analysis performed with Matlab (2015b) and Prism 7.<br>-MRI analysis was done with Paravision 6, Bruker and Prism 7.<br>-IVIS spectrum analysis performed with Living Image software and Prism 7 and 8.<br>-IgG immunohistochemistry image analysis was performed using FIJI and Prism 8.<br>-PCD data analysis was performed with Matlab (2015b) and Prism 8.<br>-LC-HR-MS/MS quantification was done using QuanBrowser 4.1.

For manuscripts utilizing custom algorithms or software that are central to the research but not yet described in published literature, software must be made available to editors/reviewers. We strongly encourage code deposition in a community repository (e.g. GitHub). See the Nature Research guidelines for submitting code & software for further information.

Data

Policy information about availability of data

All manuscripts must include a data availability statement. This statement should provide the following information, where applicable:

- Accession codes, unique identifiers, or web links for publicly available datasets
- A list of figures that have associated raw data
- A description of any restrictions on data availability

All data can be available upon reasonable request.

Field-specific reporting

Please select the one below that is the best fit for your research. If you are not sure, read the appropriate sections before making your selection.

[x] Life sciences [ ] Behavioural & social sciences [ ] Ecological, evolutionary & environmental sciences

For a reference copy of the document with all sections, see nature.com/documents/nr-reporting-summary-flat.pdf

Life sciences study design

All studies must disclose on these points even when the disclosure is negative.

Sample size	The sample size for each experimental and control condition was selected to provide sufficient statistical power to discern significant differences. Typically, non-parametric statistics were applied with a significance level of alpha < 0.05 and a power of (1-beta) > 0.8.
Data exclusions	-1 animal was excluded from Main Muscimol Delivery Experiments due to recording equipment failure.<br>-2 animals were excluded from Blank Control Experiments due to probe insertion in the wrong recording site.<br>-1 animal was excluded from FUS Stim Control because of anesthesia instability.<br>-2 animals were excluded from PCD analysis due to incorrect experimental procedures.
Replication	The experimental findings were reliably reproduced. We observed consistent and highly significant inhibition for all muscimol delivery experiments.
Randomization	Animal assignment to groups and experimental design was randomized.
Blinding	No blinding was necessary as experimental outcome was independent of any subjective assessment.

Reporting for specific materials, systems and methods

We require information from authors about some types of materials, experimental systems and methods used in many studies. Here, indicate whether each material, system or method listed is relevant to your study. If you are not sure if a list item applies to your research, read the appropriate section before selecting a response.

Materials & experimental systems

n/a	Involved in the study	
[ ]	[x]	Antibodies
[x]	[ ]	Eukaryotic cell lines
[x]	[ ]	Palaeontology
[ ]	[x]	Animals and other organisms
[x]	[ ]	Human research participants
[x]	[ ]	Clinical data

Methods

n/a	Involved in the study	
[x]	[ ]	ChIP-seq
[x]	[ ]	Flow cytometry
[ ]	[x]	MRI-based neuroimaging

Antibodies

Antibodies used	Primary antibody for immunohistochemistry: Biotinylated Goat Anti-Rat IgG (Vector Laboratories Cat# BA-9400, RRID:AB_2336202).
Validation	According to the manufacturer's website, this antibody is specific for Rat and can be used for immunohistochemistry.

Animals and other organisms

Policy information about studies involving animals; ARRIVE guidelines recommended for reporting animal research

Laboratory animals	Long Evans Rats, Female 200 gms - 300 gms
Wild animals	The study did not involve wild animals.
Field-collected samples	The study did not involve samples collected from the field
Ethics oversight	All procedures were approved by the Veterinary Office, Canton Zurich, Switzerland.

Note that full information on the approval of the study protocol must also be provided in the manuscript.

Magnetic resonance imaging

Experimental design

Design type	Structural
Design specifications	I) Localizer<br>II) Pre-Gd-DTPA injection T1 Flash Gradient Echo<br>III) Injection of Gd-DTPA<br>IV) Post-Gd-DTPA injection T1 Flash Gradient Echo<br>V) TurboRARE anatomical
Behavioral performance measures	No behavioral tasks were performed in this study

Acquisition

Imaging type(s)	Structural, Gadolinium contrast-enhanced
Field strength	7 Tesla
Sequence & imaging parameters	TurboRARE anatomical Fast Spin Echo<br><br>TE / TR: 24 ms / 4095 ms<br>NEX = 10<br>echo spacing factor = 8<br>rare factor = 8<br>slice thickness = 0.45 mm<br>matrix: 180 x 120<br>FOV 20 mm x 12 mm<br>orientation: coronal<br><br>Pre/Post Contrast Enhanced T1 Flash Gradient Echo<br><br>TE / TR: 4.5 ms / 146 ms<br>NEX = 3<br>FOV: 35 mm x 35 mm<br>matrix = 256 x 256<br>slice thickness: 0.5 mm<br>Flip Angle = 82°<br>orientation: axial
Area of acquisition	Whole brain scan was used for all animals.

Diffusion MRI ☐ Used ☒ Not used

Preprocessing

Preprocessing software	Image values were extracted using Paravision's Image Processing Tools
Normalization	The signal enhancement analysis was done similar Kobus et al: Post-Gd-DTPA enhanced contrast mean value for ROI was baseline corrected by subtracting Pre-Gd-DTPA mean of same ROI.<br>This was done for Ipsilateral vs. Contralateral ROIs respectively for AU-FUS vs. Burst-FUS.
Normalization template	No spatial normalization/transformation was used.

Noise and artifact removal | No artefact or noise removal was used.

Volume censoring | No volume censoring was used.

Statistical modeling & inference

Model type and settings | No statistical modeling or inference was used in this study.

Effect(s) tested | Experiments tested for signal enhancement post treatment

Specify type of analysis: ☐ Whole brain ☒ ROI-based ☐ Both

Anatomical location(s) | ROI center was chosen based on expected FUS Focus with reference to anatomical landmarks

Statistic type for inference (See Eklund et al. 2016) | No statistical modeling or inference was used in this study.

Correction | Corrections for multiple comparisons were not relevant.

Models & analysis

n/a	Involved in the study
☒	☐ Functional and/or effective connectivity
☒	☐ Graph analysis
☒	☐ Multivariate modeling or predictive analysis
