# Supplementary material for: Non-invasive molecularly-specific millimeter-resolution manipulation of brain circuits by ultrasound-mediated aggregation and uncaging of drug carriers
Source: Nat Commun. 2020 Oct 1;11:4929. doi: 10.1038/s41467-020-18059-7 (PMC7529901; doi:10.1038/s41467-020-18059-7)
Supplement: Supplementary file 3 — Description of Additional Supplementary Files [file 41467_2020_18059_MOESM3_ESM.pdf]

Description of Additional Supplementary Files

File Name: Supplementary Movie 1

Description: **Representative movie of aggregation and uncaging of model drug (fluorescein) from UC-carriers by AU-FUS.** UC-carriers were infused into microdialysis tubing while a sequence similar to AU_3-FUS (in vitro) was applied [for visualization purposes, t_A(ms), t_U(ms), P_U(MPa), and NOC were changed to 1.8s, 180 ms, 1MPa, and 10000 cycles, respectively].
